# Supplementary material for: Functional regimes define soil microbiome response to environmental change
Source: Nature. 2025 Jul 16;644(8078):1028–38. doi: 10.1038/s41586-025-09264-9 (PMC12390847; doi:10.1038/s41586-025-09264-9)
Supplement: Supplementary file 1 — Supplementary Information contains subsections with detailed discussions and Supplementary Table 1. [file 41586_2025_9264_MOESM1_ESM.pdf]

nature portfolio

https://doi.org/10.1038/s41586-025-09264-9

Supplementary information

Functional regimes define soil microbiome response to environmental change

In the format provided by the
authors and unedited

Supplementary Information

Contents

- A detailed description of the three functional regimes 3
- Effectiveness of chloramphenicol in preventing microbial growth in the soil 4
- Detailed mechanism of nutrient release in soils due to change in pH 5
- Effect of base cation on nutrient release in soils 6
- Recapitulating linear dynamics with monoculture experiments without carbon 7
  - Culturing protocol 7
  - Linear metabolite dynamics were recapitulated with monoculture experiments 8
- Connecting nitrate reduction rate increases to the fold change in functional biomass in Regime II 10
- Taxonomy, pH niche, and the phylogeny of the Resurgent growth strains 11
  - Determination of peak pH for each family 11
  - Constructing a phylogenetic tree with 16S rRNA sequences 12
  - Taxonomy and traits of the Regime III strains 12
- pH titration curves and physicochemical properties across soils 13
- Details of argument that long-term soil pH defines regime boundaries 15
- Evidence for long-term pH adaptation from phyla's differential response to pH perturbations 15
- Meta-analysis of functional data in previous denitrification studies in other soils 17
- Justifying widespread death as the mechanism of Regime I 18
  - Strain isolation and choosing isolates from three major phyla 19
  - qPCR quantification to measure DNA degradation at different pH levels 20
  - CFU measurements to quantify isolate's death rate in different pH levels 21
  - Comparing measured DNA degradation and death rates across varying pH levels in different strains 23
  - Cell death and DNA degradation model 24
  - Testing the odds of no-death model against sequence abundance data 25
  - Inferring the fraction of relic DNA with the model and abundance data 26

- Justifying the effective 1-biomass model despite the diversity of denitrifying taxa 27
- Prediction of nitrate dynamics from community structure 31
- Supplementary Table 1 35

A detailed description of the three functional regimes

By quantitatively distinguishing the impact of pH on the consumer side (microbial community, $\tilde{x}(0)$) and the resource side (growth-limiting nutrient, $\tilde{C}(0)$), we can ask about the mechanism behind functional adaptation during different regimes. Regime II can be called the "Nutrient-limiting" regime. Within this pH range (Fig. 3c), conditions favor the resident population of nitrate reducers; hence it allows a large indigenous nitrate reducer population to perform nitrate reduction (large $\tilde{x}(0)$). This specific range of favorable pH levels is determined by the long-term exposure to the native pH of soils (Fig. 3c). In this regime, the increase in nitrate reduction rate is determined by the biomass activity growth from the available growth-limiting nutrient. Therefore, in Regime II, the adaptive strategy employed by the nitrate-reducing population is to utilize the pre-existing resident species, which are rather robust to pH perturbations, and at the same time incrementally increase the resident's biomass activity as the resource availability changes with pH perturbations. Going back to the functional dynamics, that is the reason we see a relatively high slope of CHL+ conditions and a slight increase of denitrification rate in CHL- conditions (panels b, e of Fig. 3b), demonstrating that the resident nitrate reducers adapt to the new environment in a "nutrient-limiting" manner.

Regime III can be called the "Resurgent growth" regime. As the perturbed pH is increased from Regime II, there comes a critical pH of around 8 where the adaptive mechanism abruptly transitions. When the pH is perturbed beyond the critical point, the previously large functional biomass of the nitrate reducer population can no longer adapt and perform nitrate reduction ($\tilde{x}(0) \to 0$). On the resource aspect in Regime III, there is a surplus of limiting nutrients, and thus the system is no longer limited by C but limited by nitrate A. These two effects of short-term pH change (both on the consumer and resource aspect) set the stage for the "resurgence". A rare population, which we will investigate the composition later, appears to have a small functional biomass initially showing a flat slope in both CHL- and CHL+ conditions but later grows exponentially to exponentially deplete the nitrate in Regime III (panels c, f of Fig. 3f). This shows that in Regime III, the adaptive mechanism of the community is to rely on the rare uprising nitrate-reducing biomass to rapidly

grow in the absence of nutrient limitation.

Regime I can be called the "Acidic death" regime. This regime is at the other end (acidic) of the Nutrient-limiting regime (Regime II). As the perturbed pH is decreased from Regime II, it transitions into a regime where the system fails to adapt. The boundary pH of Regime I and Regime II is influenced by the native pH of the soil, where relatively acidic soils have a lower boundary of pH 3 or 4 and relatively neutral soils have a higher boundary of pH 5 to 6. Similar to what happens when the community enters Regime III, the unfavorable pH diminishes the indigenous nitrate-reducing activity of the soil, indicated by the flat CHL+ dynamics in panels a, d of Fig. 3b. However, unlike Regime III, the perturbed pH does not make the growth-limiting nutrient superfluous but makes it unavailable, making the divergence of CHL- and CHL+ dynamics nonexistent (panels a, d of Fig. 3a). These two effects of pH perturbation make it extremely difficult for the community to adapt to the new environment of Regime III, hence the "Acidic death" regime. Another reason we call it that is to highlight the asymmetric effect of acidic and basic perturbations, which has been seldom acknowledged in the literature.

Effectiveness of chloramphenicol in preventing microbial growth in the soil

Resistance to chloramphenicol (CHL) can be widespread in soils, and its deactivation mechanisms may break down the antibiotic over time, allowing microbial growth. For the purposes of our study, we would only be concerned by CHL resistance within the nitrate-reducing population, and only to the extent that the growth of any resistant taxa would *increase* the rate of nitrate reduction (which would then be incompatible with our model). This effect is directly measurable in our soil pH perturbation experiments and we did not observe any increase in nitrate reduction rates under CHL+ (chloramphenicol-treated) conditions. For example, if we look at nitrate dynamics during pH perturbation experiments of a soil (Fig. 1b), the slopes (derivatives) of the nitrate dynamics in CHL+ samples (red lines) are constant. In some instances, the slope slightly *decreases* at later time points, suggesting that long-term exposure to CHL may slightly decrease the nitrate reduction rates

of the denitrifying population. Crucially, we do not observe any cases where the rates increased over time (slopes becoming steeper and lines curving downward) in the presence of CHL. Furthermore, in every experiment, nitrate reduction rates in CHL- samples are consistently higher than in CHL+ samples (except Regime I). Therefore, these results confirm that CHL effectively inhibits the growth of nitrate-reducing populations.

To further validate that this inhibition was not compromised by CHL degradation, we tested a higher CHL dose and examined its impact on nitrate reduction dynamics. If CHL were breaking down sufficiently to permit microbial growth, increasing its concentration should mitigate this effect. However, we observed no significant differences in nitrate reduction between soils treated with 1000 ppm and 2500 ppm CHL (Extended Data Fig. 2c), indicating that CHL remained effective at inhibiting growth.

Detailed mechanism of nutrient release in soils due to change in pH

We turned to soil literature to develop a comprehensive nutrient release mechanism in soil under pH changes [32, 33]. Soil comprises minerals, organic matter, water, and air. Minerals and organic matter form aggregated clumps of soil particles, categorized by size into sand, silt, and clay. Clay particles, the smallest among them, consist of layers of phyllosilicates. Each layer includes tetrahedral structures of Si^{4+} covalently bonded to four oxygens and octahedral structures of continuous Mg^{2+} or Al^{3+} covalently bonded to six hydroxides [71]. Due to this chemical structure, clay particles possess numerous electrostatically charged sites, including negatively charged (oxygen atom and hydroxide) and positively charged [33] (shown as - and + sites in Fig. 5b). The clay particle's cation exchange sites are negatively charged and form ionic bonds with cations (positively charged ions), while the anion exchange sites are positively charged and bind to anions (negatively charged ions). Both cations and anions can serve as potential nutrient sources. When they are bound to the clay's exchange sites (brown section in Fig. 5b), they are protected from microbes. However, when they are released and dissolved in the pore water (light blue or pink section in Fig. 5b), they become

available to the microbial community. To understand how NaOH or HCl impacts nutrient availability, it's essential to track whether cations and anions are bound to clay particles or dissolved in the pore water.

The literature on nutrients and pH in soils proposes the following mechanism for nutrient release (Fig. 5b, see more detailed cartoon Extended Data Fig. 4j) [32, 33]. When NaOH is added to the soil solution, both Na^+ and OH^- ions (pH-mediated) act to release the anionic nutrients (case 2 of Fig. 5b). First, OH^- deprotonates ion exchange sites in the clay particles, increasing the number of cation exchange sites (- charge) and decreasing the number of anion exchange sites (+ charged), reducing the capacity of the clay to hold anions. Secondly, Na^+ can either bind to the clay particle or remain in solution [32]. The Na^+ that remains in solution increases the stability of released anions. Overall, increased OH^- increases the anionic nutrients available to the microbial community (Fig. 5b). The converse happens for HCl perturbations (case 1 of Fig. 5b).

Effect of base cation on nutrient release in soils

Nutrient release is not solely driven by OH^- ions. The base cation plays an important role, and thus whether the base cation prefers to be in the clay particle or the water solution can influence the amount of nutrients available to microbes. It is known that the bigger the size and the greater the charge of the cation, the more selectively the cation binds to clay particles. For example, the cation's binding affinity to the clay particle instead of staying in the solution is in the order of $NH_4^+ > K^+ > Na^+ > Li^+$, with divalent cations having greater binding affinity than monovalent cations (e.g., $Ca_2^+ > K^+$) [33]. When the amount of dissolved organic carbon (DOC) was measured after adding $Ca(OH)_2$ and KOH in equimolar concentrations of hydroxide, the addition of KOH resulted in a significantly higher DOC concentration [32]. This is because K^+ ions bind to clay particles with lower affinity and are more likely to remain in solution, stabilizing the released anions. This stabilization is likely due to the DOC predominantly consisting of negatively charged moieties, such as O^- groups. To check if there was a significant difference between monovalent cations,

we compared NaOH and KOH treatments for basic perturbations. We found that there was no significant difference in the nitrate utilization rates in the CHL- condition when we added the same concentrations of NaOH and KOH, indicating that the amount of limiting nutrient released was similar (Extended Data Fig. 2g), although the stabilized endpoint pH was different. As a sanity check, we further tested KCl and NaCl treatments and found that K^+, Na^+, or even Cl^- (relevant in the HCl addition) ions themselves without OH^- did not affect nutrient release (Extended Data Fig. 2g). This result agrees with previous findings [32], and shows that OH^- additions are necessary for mediating nutrient release.

Recapitulating linear dynamics with monoculture experiments without carbon

Our model and functional dynamics data suggest that the limited carbon leads to a constant rate of nitrate reduction. However, it is difficult to understand the mechanism behind this phenomenon, because if the organic carbon, an electron donor in the electron transport chain, is coupled to the reduction reaction of nitrate (terminal electron acceptor), the depletion of organic carbon will likely stop the nitrate reduction performed by the nitrate reductase enzymes. This will cause the nitrate reduction rate to be close to zero rather than the observed constant rate. As mentioned in the Discussion, one hypothesis for this phenomenon is that cells utilize endogenous carbon (electron donor) to power the electron transport chain (consuming nitrate) without needing to import carbon. To test this hypothesis, we conducted monoculture experiments with *E. coli* which performs DNRA and a known denitrifier *Pseudomonas sp.*.

Culturing protocol

Strains were pre-cultured in two stages under aerobic conditions before being transferred to denitrifying (anaerobic) conditions for phenotyping. First, wells of a sterile 24-well plate (Thermo Scientific Nunc Non-Treated Multidishes) were loaded with $1.7\,\mathrm{mL}$ of R2B medium. Wells were inoculated with *E. coli* K12 and *Pseudomonas sp.* PDM04 [58] strains from glycerol stocks stored

at $-80\,^{\circ}\mathrm{C}$. The plates were then sealed with a gas-permeable sterile membrane (Breathe-Easier, USA Scientific, 9126-2100). After sealing, the culture was incubated overnight at 0.5 rcf ($400\,\mathrm{RPM}$ in Fisherbrand Incubating Microplate Shakers 02-217-759, $3\,\mathrm{mm}$ orbital radius) and $30\,^{\circ}\mathrm{C}$ in aerobic conditions. These cultures reached saturation during this time. Second, wells of a sterile 24-well plate were loaded with $1.7\,\mathrm{mL}$ of defined media ($15\,\mathrm{mM}$ ammonium, $40\,\mathrm{mM}$ phosphate buffer with the final medium pH adjusted to 7.3, and trace metals and vitamins, as described in Ref [58]) with $25\,\mathrm{mM}$ succinate. Wells were then inoculated with $17\,\mu\mathrm{L}$ of the saturated R2B *E. coli* K12 and *Pseudomonas sp.* PDM04. After sealing, the cultures were incubated at 0.5 rcf and $30\,^{\circ}\mathrm{C}$ in aerobic conditions overnight. These cultures reached saturation during this time. Saturated defined media (DM) cultures were washed and normalized to a desired optical density (measured at $600\,\mathrm{nm}$) via dilution into pH 7.4 phosphate-buffered saline ($8\,\mathrm{g/L}$ H_2O, $0.2\,\mathrm{g/L}$ KCl, $2.68\,\mathrm{g/L}$ $Na_2HPO_4{\cdot}7H_2O$, $0.24\,\mathrm{g/L}$ KH_2PO_4).

Wells of a sterile 96-deep well plate (Axygen PDW20C) were loaded with carbon-free $1.2\,\mathrm{mL}$ DM supplemented with 2 mM sodium nitrate which had been allowed to equilibrate in the anaerobic glovebox. These wells were inoculated in the glovebox with $12\,\mu\mathrm{L}$ of OD-normalized aerobic pre-cultures, resulting in starting ODs of 0.1 and 0.01. Additional wells were left blank as no-growth controls. Plates were sealed with a gas-permeable sterile membrane. Cultures were incubated at $30\,^{\circ}\mathrm{C}$ and shaken at $950\,\mathrm{RPM}$ (Fisherbrand Incubating Microplate Shakers 02-217-759 or Talboys Professional 1000MP, 3 mm orbital radius) for $72\,\mathrm{h}$. Optical densities of initial and endpoint anaerobic pre-cultures were measured using $300\,\mu\mathrm{L}$ of cultures in 96-well optical plates. Nitrate and nitrite concentrations were assayed over time via manual sampling and subsequent Griess assay and vanadium (III) chloride reduction via the protocol described in Ref. [58].

Linear metabolite dynamics were recapitulated with monoculture experiments

Both *E. coli* K12 and *Pseudomonas sp.* PDM04 strains were able to reduce nitrate even without carbon in the culture media (Extended Data Fig. 2e). The reduction rate was negligible for *E.*

coli strain at a starting OD600 of 0.01 (optical density at 600nm). However, for the denitrifier *Pseudomonas sp.* PDM04 at a starting OD600 of 0.01, not only was the rate of nitrate reduction comparable to what we observed in soils at the Nutrient-limiting regime (Regime II), but the reduction dynamics were strikingly linear. This result directly demonstrates that nitrate reduction can proceed even when carbon is not exogenously available. Our observations are consistent with this hypothesis that the cells utilize endogenous carbon (like protein or nucleic acids, see Discussion) and oxidize this carbon to provide electrons (NADH) to reduce nitrate to nitrite. If the nitrate reduction rate had increased, it would have meant that the biomass activity increased. The nitrate reduction rates did not increase throughout the experiment (top panel in Extended Data Fig. 2e). This supports the idea that cells are using nitrate to maintain biomass activity. Consistently, final OD600 measurements did not detect any significant increase from the initial OD600 as expected. These results suggest that the functional biomass in soils can utilize nitrate at a constant rate, even after external carbon is no longer available. Note our model assumes this to be the case (Fig. 2).

In the monoculture experiment, we observed biphasic behavior in the high initial OD600 condition. This phenomenon is challenging to interpret. In the starting OD600 of 0.1, the initial slopes of nitrate reduction dynamics are constant, then after approximately one day, the rates decreased and remained constant (we will call this “late slope”) until the end of the experiment (bottom panel of Extended Data Fig. 2e). The linear dynamics observed in the “late slope” again still recapitulate the linear dynamics we observed in the soils in the Nutrient-limiting regime, where the microbes could be using nitrate and internal carbon to generate maintenance energy. In these monoculture experiments, it was difficult to understand what determines the late slope values. One hypothesis consistent with the use of endogenous carbon is that these strains switch the internal carbon pool that is being degraded (e.g., protein to nucleic acids). But confirming this awaits further studies. The initial slopes can be roughly explained by their starting biomass activity (Extended Data Fig. 2f), where the initial slope for the OD600 0.1 condition was roughly 10 times greater than that for the OD600 0.01 condition.

Connecting nitrate reduction rate increases to the fold change in functional biomass in Regime II

To connect $x(t)$ to the sequencing data, we investigated whether the increase in functional biomass corresponds to increases in nitrate reduction rates. In Extended Data Fig. 4d, the fold increase of total biomass (calculated as the absolute abundance in CHL- relative to CHL+ conditions) exhibited a linear relationship with the amount of base added (up to $\sim$25 mM OH^-), corroborating our proposed nutrient release mechanism in Regime II. However, since not all biomass participates in nitrate reduction, we need to identify the subset of the total biomass that actively performs nitrate reduction, which we call "functional biomass", the $x(t)$ variable in our model. Our goal was to identify the fraction of biomass responsible for nitrate reduction.

To assess the deviation that occurs when assuming all total biomass performs nitrate reduction, we plotted the fold increase in total biomass against the fold increase in nitrate reduction (CHL- vs. CHL+). As expected, the fold change in total biomass showed poor alignment with the fold increase in nitrate reduction rate, showing too little biomass change to account for the observed rate change (falling below the 1:1 line in Extended Data Fig. 4e). This motivated us to identify the functional biomass.

To find the functional biomass that performs nitrate reduction, we used differential abundance analysis to statistically determine which amplicon sequence variants (ASVs) were significantly enriched in each pH perturbed condition in CHL- condition compared to the CHL+ counterpart serving as a baseline of no growth (see Methods). Among these significantly enriched ASVs, we removed ASVs that were also significantly enriched in 0 mM nitrate control conditions comparing CHL- and CHL+ samples under no pH perturbation (dark grey, No-nitrate responders in Extended Data Fig. 4g,h). We regarded them as false-positive nitrate reducers, possibly growing through fermentation without requiring nitrate. We calculated the functional biomass by aggregating the absolute abundance of the remaining ASVs for each condition (sample). When we computed the fold increase of functional biomass from T0, we observed improved alignment between biomass

fold increase and the rate increase along the 1:1 line in the scatter plot for each soil (Extended Data Fig. 4h).

While some soils showed very close agreement between the inferred increase in functional biomass and increases in nitrate reduction rate (Soil 5 and 11 in Extended Data Fig. 4h), for other soils the relationship was not quantitative. This discrepancy likely arises from the fact that we inferred the taxa that are not nitrate reducers from slurries where the pH was not perturbed. Thus, the no-nitrate responders may be distinct as pH is perturbed, and this may increase errors in our inference of changes in functional biomass.

Taxonomy, pH niche, and the phylogeny of the Resurgent growth strains

Determination of peak pH for each family

To elucidate the pH niche of each family in Extended Data Fig. 6f, we analyzed the relative abundance of the chloramphenicol-untreated (CHL-) conditions of ASVs identified as being enriched in different pH levels (see Differential abundance analysis, Methods). Due to the challenge of visualizing a large number of ASVs, we aggregated the relative abundance of ASVs in the same family for each sample to visualize the data at the family level. We took three steps to get the representative relative abundance of each family across the perturbed pH level. First, for each family, we computed the median of the relative abundance from three replicate samples. Now, for each family, we have 130 relative abundance values from 10 different soils with 13 different pH perturbed levels across pH 3 to 9. Second, for each family, we binned these relative abundance values by their corresponding perturbed pH from lowest to highest, making each pH bin have at least 2 relative abundance measurements. Third, we computed the median of the relative abundance values in each pH bin. For each family, we can now plot the median relative abundance values across perturbed pH (mean pH of each pH bin). We assigned peak pH values for each family by finding the pH (mean pH of pH bin) at which the family has its maximum median relative abundance across all pH bins. We ordered all families according to their peak pH values from lowest to highest.

To reduce the number of families to plot, we included families with a median relative abundance greater than 0.002 at the second peak pH bin, resulting in 89 families. After ordering the 89 families by their peak pH from lowest (top, dark blue) to highest (bottom, yellow), we generated a ridge plot where the x-axis was the perturbed pH (mean pH of pH bin) and the height was the median relative abundance of each bin (Extended Data Fig. 6f). The height in each row was normalized by each family's maximum median relative abundance. The family with peak pH over 8 was mostly Bacillota phylum (Bacillaceae, Clostridiaceae, Paenibacillaceae, Caloramatoraceae, Peptostreptococcaceae, Lachnospiraceae), other than the Yersiniaceae family, which was Pseudomonadota.

Constructing a phylogenetic tree with 16S rRNA sequences

To see whether there was phylogenetic convergence among families with similar pH niches, we used the 16S rRNA sequences of the ASVs to construct a phylogenetic tree (Extended Data Fig. 6h). We used the ASVs that belonged to the 89 families in the previous pH niche analysis. We selected one ASV with the largest relative abundance from each family to represent the family and used its 16S rRNA sequence to construct the phylogenetic tree. The phylogenetic tree was constructed by approximating the Maximum likelihood tree with the General time reversible model in FastTree ver. 2.1.9 [72]. The tree was plotted using the plot.phylo function in ape package in R, each node (labeled with the classified genera or species name) colored by its peak pH. We observed that ASVs with similar pH niches did not cluster phylogenetically and were dispersed throughout the phylogenetic tree (Extended Data Fig. 6h).

Taxonomy and traits of the Regime III strains

To identify the specific taxa accountable for the emergence of Regime III at a finer taxonomic level, we conducted a differential abundance analysis that statistically determined which Amplicon sequence variants (ASVs) were significantly more abundant in Regime III CHL- samples, compared to CHL+ samples under same perturbed conditions (see Methods). Then, we aggregated the relative abundance of these differential ASVs (i.e., Regime III strains) to assess their contribution to the

emergence of Regime III. Notably, their abundance began to rise between pH 7-8 (Extended Data Fig. 6d), which aligns with or slightly precedes the transition between Regime II and III (Extended Data Fig. 6e). The analysis revealed that 8 families belonging to Bacillota (Bacillaceae, Paenibacillaceae, Clostridiaceae, Caloramatoraceae, Peptostreptococcaceae, etc.) and 2 families belonging to Pseudomonadota phylum (Legionellaceae and Yersiniaceae) were significantly enriched in Regime III (Extended Data Fig. 6c). At the genus level, Bacillus, Clostridium, Paenibacillus, and others were identified as the primary contributors to Regime III.

To understand why these families better adapt to higher pH and high carbon conditions, we sought to find distinct features of the Regime III families that differentiated them from others. First, regarding the high pH environment, we ordered all families by their pH niche (peak pH), the pH where each family had its highest relative abundance across different perturbed pH levels (Extended Data Fig. 6f). We indeed found that the Regime III families had distinct pH niches compared to other strains, having high relative abundance in basic pH (over 8) and in some cases acidic pH (less than 4), but remained rare ($< 0.1\%$) in the mid-range of pH 4-8. One can speculate that their ability to survive and persist in extreme pH perturbations (see Extended Data Fig. 7a) may be because many taxa in the phylum Bacillota are spore-forming bacteria species [73]. Second, we inferred the genotypes of each Family's ASVs using PICRUSt2 (see Methods: "Genotyping enriched ASVs with PICRUSt2"). We found that Bacillaceae, one of the Regime III families enriched in extreme basic perturbations, was responsible for the enriched *nrfAH* genes used for DNRA in slurry samples (highlighted in bold in Extended Data Fig. 6g). This may be pertinent to why Regime III taxa are enriched in high carbon conditions because DNRA is favored over denitrification in high C:N ratio environments [29].

pH titration curves and physicochemical properties across soils

We've constructed pH titration curves for the 20 soils from different native pH levels (see Methods, Extended Data Fig. 8n). Because we titrated both in acid and basic directions with H^+ and OH^-,

respectively, we unified the x-axis to OH^- (m mol) by shifting the curves to the right by 0.2 m mol, ensuring each curve starts at 0 m mol OH^-. We then fitted the pH titration curves with a logistic function with 4 parameters (a, x_{mid}, b, c) as below (visualized in Extended Data Fig. 8o):

$$pH = \frac{a}{1 + e^{\frac{-(x - x_{mid})}{b}}} + c \tag{4}$$

Parameter x_{mid} strongly correlated with soil's native pH level (R^2 = 0.8, Extended Data Fig. 8q), while parameter c (y-intercept) and a+c (asymptotic y value), scaling parameter (b) stayed mostly constant across soils with different pH levels. This indicates that the titration curve's general shape is similar for all soils, but the titration curve shifts horizontally depending on the soil's original pH level.

Soil's native pH, which determines the horizontal shift of the titration curves, was strongly correlated to the cation exchange capacity (CEC, milliequivalent charge / 100g) (R^2 = 0.88, Extended Data Fig. 8q). This was expected because soils with higher CEC will have a greater number of negative charges in the clay particles and hence are more likely to adhere to protons. This will result in fewer free protons in the soil pore water, and thus result in more basic pH levels. In the literature, CEC is reported to be determined by soil's clay particles and its organic matter, because CEC is proportional to how much negative charge the soil has on the aggregate's surface. However, in our dataset, the percent clay and organic matter did not correlate strongly with CEC (Extended Data Fig. 8q, see Fig. 6a for percent clay). CEC appeared to be determined by Ca^{2+} ion concentration in the soil and not by other cations (Mg^{2+}, K^+, Na^+). Soil pH was inversely correlated with S, P, Al, and Fe concentrations, which can either be the cause or result of the soil's pH (Extended Data Fig. 8q). In sum, we can attribute the horizontal shift of the pH titration curves to their varying native soil pH levels, which is potentially determined by the CEC and the Ca^{2+} ion concentrations (see summarized diagram in Extended Data Fig. 8p).

Details of argument that long-term soil pH defines regime boundaries

Here, we present evidence supporting the section "Long-term soil pH defines regime boundaries" from the main text. The shape of the titration curve was similar across all soils (Extended Data Fig. 8n), showing a plateau at low and high pH with a nonlinearity in between. Acidic soils are more strongly pH-buffered than the neutral soils (Extended Data Fig. 8b, Extended Data Fig. 8n). Thus, for similar levels of acid addition, neutral soils would experience *a larger drop in pH* than acidic soils ($\Delta\text{pH}^{acidic} < \Delta\text{pH}^{neutral}$, Extended Data Fig. 8a,b). We speculate that this makes communities in acidic soils less tolerant of acidic pH fluctuations, as they are less likely to experience large reductions in pH. This reasoning would help explain the observation that acidic soils transition from Regime II to I after a smaller perturbation in pH than neutral soils (Extended Data Fig. 8e, $\Delta\text{pH}^{acidic} < \Delta\text{pH}^{neutral}$). As a result, plotting the pH at the Regime II to I transition against the native pH gives a line with slope <1 (Extended Data Fig. 8e, bottom dashed line), where a line of slope 1 would indicate that entry into Regime I requires a constant change in pH.

In contrast, we find that soils transition from Regime II to III when carbon is in excess. From Fig. 5, we know that carbon is released in proportion to the NaOH added to the slurry (Extended Data Fig. 8c). Accordingly, we find that a constant addition of NaOH drives the transition from Regime II to III. However, due to the shape of the titration curves as seen in Extended Data Fig. 8d, for a constant base amendment, more neutral soils reach higher pH (dashed line Extended Data Fig. 8e). Therefore, as expected from the titration curves, more basic soils transition to Regime III at higher pH (Extended Data Fig. 8e).

Evidence for long-term pH adaptation from phyla's differential response to pH perturbations

We observed that long-term pH variation (different native soil pH) shifts the pH boundaries between functional regimes (Extended Data Fig. 8a-e). To see if those shifts of pH boundary can be explained by taxa's differential response to perturbed pH, we further asked whether the pH

values where the abundances of taxa (Bacillota, Bacteroidota, and Pseudomonadota) exhibit large changes also agree with the boundaries between regimes determined solely by nitrate utilization dynamics (Fig. 3). We observed the growth folds of taxa for the transition from Regime II to Regime III and the survival folds of taxa for the transition from Regime II to Regime I. Growth folds were computed by endpoint absolute abundance ratio of $\mathrm{Abs}_{CHL-}/Abs_{CHL+}$ (chloramphenicol untreated/treated conditions) and survival folds were computed by absolute abundance ratio of Abs_{CHL+}/Abs_{T_0}, representing taxa's endpoint absolute abundance in CHL+ conditions compared to the initial time point (T_0) for each perturbed pH level.

To understand the transition to the Acidic death regime (Regime I), we observed the survival folds of Pseudomonadota and Bacteroidota phyla across perturbed pH levels. Then, we set an identical survival fold threshold for all soils to compute the pH at which the survival fold goes below that threshold during acidic perturbation. We used two distinct definitions to choose a threshold for the survival fold. The first was a definition of "dying" where the taxa's abundance started to decline in abundance compared to T_0 (survival fold threshold < 1). The second was a definition of "dead" where the taxa's abundance was close to 0 (survival fold threshold $\rightarrow 0$). For each of these definitions, the pH transition points were plotted (Extended Data Fig. 8f with the first "dying" definition and Extended Data Fig. 8g with the second "dead" definition) and compared to the trends of functional regime boundaries (transition from Regime II to I). Employing the 'dying' definition with Pseudomonadota, Bacteroidota allowed us to recapitulate the phenomenon observed in the functional data, where the fitted slope ($= 0.56 \pm 0.09$) of Boundary I-II was less than 1 (Extended Data Fig. 8f). This suggests that these phyla in the relatively neutral soil are more tolerant of larger ΔpH change until they start to die than those in acidic soils (Extended Data Fig. 8b). Because the fitted slope is greater than 0, this also means that these phyla in relatively acidic soils can tolerate lower pH conditions than those in neutral soils, which suggests adaptation to low pH, although more complex ecological mechanisms cannot be ruled out. The 'dead' definition threshold resulted in a flat slope ($= 0.11 \pm 0.08$) close to 0 (Extended Data Fig. 8g). This suggests that, despite long-

term adaptation to varying native soil pH levels, these taxa have similar pH thresholds at which complete death occurs. Experiments measuring death rates of isolates as a function of pH support this basic picture with differential death rates around pH= 4 and uniform rapid death around pH= 3 (Extended Data Fig. 7a).

Similarly, to understand the transition to the Resurgent growth regime (Regime III), we observed the growth folds of Bacillota phylum across perturbed pH levels. Then, we applied an identical growth fold threshold for all soils to compute the pH at which the growth fold goes above the threshold during basic perturbations. These pH transition points were plotted (Extended Data Fig. 8f,g) and compared to the trends of functional regime boundaries (transition from Regime II to III). Consistent with the trend of functional regime boundary II-III (Extended Data Fig. 8e), the abundance of Bacillota began to increase at higher pH values as the native soil pH increased. Note that the NaOH amount, and consequently, the quantity of released carbon, remains approximately constant at the Regime II-III boundary, suggesting that the increase of Bacillota increases at higher pH in more neutral soils might not be explained by additional nutrients. This result suggests the Regime III strains within each soil are locally adapted to the pH conditions of that soil.

Meta-analysis of functional data in previous denitrification studies in other soils

To extend the generality of our findings, we conducted an in-depth meta-analysis of 9 denitrification studies spanning 70 years (soil and study information in Table S2), covering diverse soil types and global locations (Fig. 6a). We re-analyzed the data from a study on three Czech soils [35] to confirm the existence of functional regimes during pH perturbations (Fig. 6c). Simek *et al.* [35] measured the early denitrification rate within 90 minutes (Denitrification enzyme activity, DEA) and the late denitrification rate throughout the next 48 hours (Denitrification potential, DP). The short-term DEA dynamics closely followed a linear trend, so we performed a linear fit to determine the denitrification rate and plotted the slopes as the x-axis in Fig. 6c as a proxy for indigenous biomass activity $\tilde{x}(0)$. The long-term DP dynamics were non-linear, so we calculated the slopes

at each time step and identified the fastest denitrification rate. To infer available limiting nutrient $\gamma\tilde{C}(0)$, we calculated the change between early and late denitrification rates, which are on the y-axis of Fig. 6c. The relative abundances of phyla in Fig. 6e were directly extracted from the sequencing data of Anderson *et al.*[30]. For the DEA measurement in Fig. 6f, with the exception of Simek data in Fig. 6c discussed above, all other data points were directly extracted from the other three studies [27, 36, 37]. The N dynamic data points in the left panel of Fig. 6g were extracted from the total N measurements by the study in the UK [39]. The N dynamics in the right panel of Fig. 6g were extracted from N_2O and N_2 measurements by the study in Norway [38]. This meta-analysis was reported according to the Preferred Reporting Items for Systematic Reviews and Meta-Analyses (see PRISMA statement in SI).

Justifying widespread death as the mechanism of Regime I

We showed with sequencing data that the potential mechanism underlying Regime I is widespread death at low pH (Extended Data Fig. 7a). However, because we measure taxa abundance by sequencing DNA to infer death, there is a possibility that the consistent drop in survival folds during acidic perturbations (Extended Data Fig. 7a) may be due to increased degradation of extracellular DNA of dead microorganisms in the soil (known as relic DNA), rather than death due to low pH. Soils are known to harbor large amounts of extracellular DNA from dead microorganisms ($\sim$40% out of sequenced DNA) in some samples [34]. Nucleic acids are less stable and more prone to degradation at acidic pH than at neutral pH due to proton-mediated depurination [74]. Therefore, we experimentally and quantitatively tested whether this decrease in sequence-based abundance during acidic perturbation is an artifact derived solely from the degradation of relic DNA in low pH rather than death. To do so, we:

1. Under varying pH conditions we measured the degradation rates of DNA from isolates belonging to three phyla.

2. Measured the death rates of the same strains by performing time-series CFU assays in mono-

cultures in defined media across pH levels.

3. Built a simple model (5) of cell death and DNA degradation to predict the decrease in sequence-based abundance measurements we expect given the measured DNA degradation with and without cell death. The model shows that the sequence-based abundance data cannot be explained in Regime I **without cell death**.

Strain isolation and choosing isolates from three major phyla

We aimed to isolate strains that belonged to the three major phyla whose abundance correlated with the three regimes (Fig. 4): Pseudomonadota, Bacteroidota, and Bacillota. For isolation, we thawed endpoint slurry samples from pH perturbation experiments stored in 25% glycerol in $-80\,^{\circ}\mathrm{C}$, streaked onto 1/10× tryptic soy agar (TSA) plates and 1/10x Reasoner's 2A (R2A) plates with pH adjusted to correspond to the endpoint pH measurement of the slurry sample. The plates were incubated at $30\,^{\circ}\mathrm{C}$ and were checked for new growth every 24 hours. Colonies were restreaked twice onto 1/10x TSB plates or until pure. After culturing pure colonies in 1/10× tryptic soy broth (TSB), we extracted genomic DNA using the DNeasy Ultraclean Microbial Kit (Qiagen, Hilden, Germany). To identify unique strains, we did Sanger sequencing on the 16S rRNA region amplified by 27F and 1492R primers and performed BLAST on the sequence to the NCBI database, obtaining a strain collection library of $\sim$60 unique strains. To confirm they are denitrifiers, we tested their ability to reduce nitrate in the anaerobic chamber in succinate-defined media (SDM) as described previously [58]. We compared their full 16S rRNA sequences to the ASV's V3-V4 region sequences classified as Regime II and Regime III strains from the previous differential abundance analysis (Methods). For a representative Pseudomonadota strain, we selected isolate JW70530, a *Massilia* sp., which was classified as the Regime II strain. For a representative Bacillota strain, we chose JW50604, a *Paenibacillus* sp. which was classified as a Regime III strain. We were not able to isolate strains from the phylum Bacteroidota. Therefore, for a representative Bacteroidota strain, we used isolate sic0106, a *Chitinophaga* sp. from our denitrifier strain collection from a previous

study [58], because many Bacteroidota ASVs enriched in Regime II were from the *Chitinophaga* genus. For the following qPCR and CFU assays, we used these three strains and called them *Massilia* (Pseudomonadota), *Chitinophaga* (Bacteroidota), and *Paenibacillus* (Bacillota) strains.

qPCR quantification to measure DNA degradation at different pH levels

For three strains representing different phyla, we cultured each in 12mL of 1/10× TSB to extract genomic DNA (gDNA) using the DNeasy Ultraclean Microbial Kit (Qiagen, Hilden, Germany). Then, we quantified the gDNA concentration with Qubit DNA quantification assay kit (Thermo Fisher, MA, USA) and diluted it to 100 dsDNA $\mathrm{ng/\mu L}$. To measure varying DNA degradation rates at different pH levels, we made 50 mM phosphate potassium buffers of pH 3, 3.5, 4, 5, 6, and 7 by mixing solutions of 50 mM phosphoric acid, monopotassium phosphate, and dipotassium phosphate in different ratios. Then, we incubated $10\,\mathrm{ng/\mu L}$ of gDNA in a total volume of $120\,\mathrm{\mu L}$ of different pH buffer conditions in triplicate at $25\,^{\circ}\mathrm{C}$ for 4 days, sampling at 0hr, 1hr, 2hr, 4hr, 8hr, 14hr, 25hr, 52hr, 80hr of incubation, stored at $-20\,^{\circ}\mathrm{C}$. To measure the amount of DNA, we performed PCR (qPCR) with the QuantStudio Pro Real-Time PCR system (Applied Biosystems, CA, USA) using the same primers for our 16S rRNA amplicon sequencing so that we could better recapitulate the abundance measurements we performed in slurry experiments. We sampled $2\,\mathrm{\mu L}$ of each time point from each replicate well and did qPCR with FastStart Essential DNA Green Master mix (Roche, Basel, Switzerland) in a total reaction volume of $20\,\mathrm{\mu L}$ with 150nM of forward primer 341-b-S-17 (CCTACGGGNGGCWGCAG) and reverse primer 785-a-A-21 (GACTACHVGGGTATCTAATCC).

Due to polymerase amplification efficiency varying in different pH phosphate buffers, we constructed standard curves with varying gDNA concentrations (triplicate of 10, 5, 2.5, 1.25, 0.625 gDNA $\mathrm{ng/\mu L}$) for every pH buffer condition for all three strains using one separate 96 well plate for each taxon (R^2 ranging from 0.97 to 0.99). We did linear regression on the known gDNA concentrations against the log10-transformed quantification cycle (Cq) outputs from the Design &

Analysis Software 2.8.0 provided by the qPCR system manufacturer. This allowed us to acquire the standard curve's slope, necessary to convert Cq values to DNA concentrations from experimental samples in other plates. Because the normalized fluorescence emission (ΔRn) cutoff used to determine Cq values varies between 96-well plate runs, we utilized the Cq values of the T0 samples ($10\,\mathrm{ng}/\mu\mathrm{L}$) of each taxon and each pH buffer to complete the standard curve equation for computing the DNA concentrations from Cq value outputs of other time points in the same plate. We observed positional effects (i.e., brightening effect in the center row of the 96 well plate), and thus included $10\,\mathrm{ng}/\mu\mathrm{L}$ samples of gDNA in Milli-Q water for every row of our qPCR plate to control for this effect. To measure the DNA degradation rate constant (δ) in equation (5), we performed linear regression on the log10-transformed non-zero DNA concentration time points of each taxon and each pH buffer condition using fitted slopes as δ (Extended Data Fig. 7c).

CFU measurements to quantify isolate's death rate in different pH levels

To learn the death rates of the three different strains (*Massilia* (Pseudomonadota), *Chitinophaga* (Bacteroidota), and *Paenibacillus* (Bacillota)) as a function of pH, we incubated them in defined pH buffered media lacking nutrients required for growth and measured the number of viable cells over time by plating and counting colony-forming units (CFUs). Before performing the death assay, the strains were pre-cultured through an aerobic growth stage followed by an anaerobic growth stage for strains to adapt to sparse-nutrient anaerobic conditions gradually. In the aerobic growth step, strains were cultured under aerobic conditions in 1/10x TSB for 2 days from glycerol stocks, then passaged 1:100 into succinate defined medium, designed for cultivation of denitrifying bacteria [75], and grown until the medium became visibly opaque (3 days for *Massilia* and *Paenibacillus*, 5 days for *Chitinophaga* strain). The succinate-defined medium (SDM) contained 25 mM succinate as the sole non-fermentable carbon source, 15 mM ammonium as the assimilatory nitrogen source, and a 40 mM potassium phosphate buffer with pH adjusted to 7.3. Trace metals and vitamins were also added. After the aerobic pre-culture step, each strain was normalized to OD600 = 1, passaged

1:100 into SDM (4 mM succinate, 1 mM nitrate, pH 7), incubated for 3 days at 30 °C inside the anaerobic chamber. In the 4 mM succinate-defined medium, we added 1 mM nitrate to enable the strains to utilize the non-fermentable succinate as a carbon source through anaerobic respiration. After the anaerobic pre-culture step, we normalized each strain to OD600 = 0.05, passaged 60 µL into 940 µL of SDM (4 mM succinate, no nitrate) of 6 different pH levels (pH of 3, 3.5, 4, 5, 6, and 7 with 40 mM potassium phosphate buffer) in triplicate, and incubated them for measuring death rates at 30 °C and 950 rpm in the anaerobic chamber. All defined media used in the anaerobic chamber were degassed for at least 12 hours prior to incubation.

We sampled 10 µL for each strain and condition every time point spanning 4 days of incubation: 0hr, 1hr, 3hr, 7hr, 13hr, 25hr, 56hr, 74hr, 100hr. These samples were serially tenfold diluted aiming to obtain suspensions containing approximately between 10 to 100 CFUs. In a biosafety cabinet, 10 µL of each dilution series was pipetted onto agar plates using a multi-channel pipet and streaked by tilting the plate until the droplets reached the opposite edge. After allowing the plates to dry, we incubated them at 30 °C for 2 days under aerobic conditions. CFUs were manually counted using the click counter tool in Fiji software [76]. For the agar plates, we used R2A agar because *Massilia* strain was not able to form any colonies in 1/10x TSA agar plates at this step. Although the *Chitinophaga* strain formed colonies on R2A plates at T0, no colonies were observed after 1 day, likely due to a high death rate even under the pH 7 incubation condition. We hypothesized that the prolonged pre-culture steps for gradual adaptation were not conducive to the viability of the *Chitinophaga* strain during the final anaerobic incubation step. We shortened the pre-culture steps by first culturing the *Chitinophaga* strain aerobically in 1/10x TSB for 2 days from glycerol stocks, washing the culture three times with 0.9% saline, normalizing to OD600 = 1, and then passaging 1:100 into the potassium phosphate buffer at various pH levels. Using the same method, we plated the time-series serially diluted samples onto 1/10x TSA plates and measured CFUs. To measure the cell death rate constant d in equation (5), we performed linear regression on the log10-transformed CFU time points (greater than 10^3 CFU/mL) of each taxon and pH condition, the fitted linear slopes

being death rate d (Extended Data Fig. 7d). For the *Chitinophaga* strain, because the cell death was extreme during the first hour of incubation and then stabilized to a constant rate thereafter, we used time points starting from T1 ($\sim$1 hour) to fit the line.

Comparing measured DNA degradation and death rates across varying pH levels in different strains

For DNA degradation, all three strains from different phyla had relatively identical DNA degradation rates across pH levels tested from 3 to 7 (Extended Data Fig. 7e). Sequences with more thymine are reported to degrade much faster by acid (depurination) than sequences with less thymine [74]. The identical degradation rates may be due to the 16S rRNA region being conserved, and thus strains' amplified DNA region having similar nucleotide composition. In the course of 4 days, we detected no degradation of DNA in pH 5, 6, and 7 of all three strains. For pH 4, only $\sim$10% of DNA degraded per day. For pH 3.5, $\sim$20% to $\sim$30% of DNA degraded per day. But for pH 3, $\sim$99.8% of DNA degraded per day. Because DNA degrades relatively slowly at pH 4 and 3.5 compared to pH 3, these pH levels are ideal for testing for cell death in Regime I. If sequencing data in Regime I shows a significant decrease in the absolute abundance of taxa compared to the null expectation based on DNA degradation from this experiment, it indicates the existence of cell death.

For death rates d, we saw more strain-by-strain variation with death rates of all three strains increasing at lower pH levels (Extended Data Fig. 7d). However, *Paenibacillus* strain did not recapitulate the pH response of Bacillota from the sequencing data where its absolute abundance did not decrease at lower pH. We hypothesized that its pH-resistant, spore-forming capabilities in soil cannot be reproduced under laboratory media conditions. Interestingly, death rates were consistently faster than DNA degradation rates across all strains and pH levels (Extended Data Fig. 7e). The baseline death rate at neutral pH under media conditions was not 0 for all strains (Extended Data Fig. 7h). Even if we take into account this baseline by subtracting the minimal death rate across pH for each strain, the death rate is much greater than the DNA degradation rate (Extended Data Fig. 7f). To quantitatively answer this question of the existence of widespread death

in Regime I, we constructed a simple model of DNA degradation and cell death, used the DNA degradation rate constants measured from the experiment, and tested whether DNA degradation alone, without cell death, could explain our sequence abundance data.

Cell death and DNA degradation model

Consider a model of two variables: biomass $X(t)$ (DNA ng) and free extracellular DNA $D(t)$ (ng) in the soil, which evolves over time (t, day). The measured biomass M(t) (DNA ng) from 16S rRNA amplicon sequencing is the sum of microbial functional biomass $X(t)$ and free extracellular DNA $D(t)$. The ordinary differential equations (ODEs) can be expressed as:

$$\begin{aligned} \dot{X}(t) &= -dX(t), \\ \dot{D}(t) &= \alpha dX(t) - \delta D(t), \\ M(t) &= X(t) + D(t) \end{aligned} \tag{5}$$

, where d is the biomass death rate constant (per day), δ is the DNA degradation rate (per day), and α is the amount of DNA released from dead cells to the soil per biomass (DNA ng/biomass). The first equation of (5) represents the biomass death rate, which is determined by the microbial biomass (X, DNA ng) and cell death rate (d, 1/day). The second equation of (5) represents the DNA degradation rate, which is determined by the degradation of DNA at rate δ (1/day) and the production of extracellular DNA released by dead biomass at rate $\alpha dX(t)$ (DNA ng/day) (Extended Data Fig. 7g).

Solving the differential equations (5) will give solutions of:

$$\begin{aligned} X(t) &= X(0)e^{-dt}, \\ D(t) &= \frac{\alpha dX(0)}{\delta - d}e^{-dt} + (D(0) - \frac{\alpha dX(0)}{\delta - d})e^{-\delta t}, \\ M(t) &= (\frac{\alpha d}{\delta - d} + 1)X(0)e^{-dt} + (D(0) - \frac{\alpha dX(0)}{\delta - d})e^{-\delta t}, \\ &= (\frac{\alpha d}{\delta - d} + 1)(1 - f)M(0)e^{-dt} + (fM(0) - \frac{\alpha d(1 - f)M(0)}{\delta - d})e^{-\delta t} \end{aligned} \tag{6}$$

, where f is the fraction of relic extracellular DNA out of total DNA extracted from the soil at T0.

$$f = \frac{D(0)}{X(0) + D(0)} \tag{7}$$

Testing the odds of no-death model against sequence abundance data

Our goal was to quantitatively ask whether widespread death is present in Regime I despite DNA degradation occurring at low pH. Therefore, we tested whether DNA degradation alone, without cell death, could explain our sequence abundance data. If the no-death model ($d = 0$), given the measured DNA degradation rate δ, cannot statistically explain the abundance distribution, this would strongly indicate that death exists and is required to explain the sequence abundance change during Regime I. The no-death model is acquired when we set $d = 0$ as the following equations:

$$\begin{aligned}
\dot{X}(t) &= -dX(t) = 0, \\
\dot{D}(t) &= \alpha d X(t) - \delta D(t) = -\delta D(t), \\
M(t) &= X(t) + D(t) = X(0) + D(0)e^{-\delta t}, \\
&= (1-f)M(0) + fM(0)e^{-\delta t}
\end{aligned} \tag{8}$$

For δ values, we will use the DNA degradation rate we measured with the strain's gDNA for each representative phylum and each pH condition. We also know $M(0)$ from the sequence-based absolute abundance data at T0. Therefore, the prediction of taxa abundance measured by sequencing, $M(t)$, from the no-death model would depend only on f, the fraction of relic DNA. We can set f to either 0 (0% relic DNA), 0.5 (50% relic DNA), or 1 (100% relic DNA), where $f = 0.5$ could be a more realistic scenario [34]. In these scenarios, we simulated $M(t)$ for every phylum and pH condition (Extended Data Fig. 7h) after normalizing $M(0)$ to 1.

Next, to compare these no-death model $M(t = endpoint)$ predictions with the observed distribution of the endpoint absolute abundance from the CHL+ (chloramphenicol-treated) samples, we looked at the absolute abundance at the family level that corresponded to the three phyla (Pseudomonadota, Bacteroidota, and Bacillota).To minimize the impact of noise from the larger variability associated with low-abundance taxa, we filtered out families with an initial absolute abundance smaller than 0.001. We used CHL+ samples because we would like to only observe the effect of death and not growth. Using CHL+ samples from all 10 CAF soils, we binned the pH-perturbed endpoint samples into pH 3, 3.5, 4, 5, 6, and 7 bins, if their endpoint pH was ± 0.2 within the corre-

sponding pH bin. Then, to plot the abundance distribution of different families in the same phylum together in a histogram, we divided each family's endpoint absolute abundance with the initial absolute abundance (normalized $M(t = endpoint) = M(T9)/M(0)$ (Extended Data Fig. 7i).

To quantify how much the no-death prediction deviates from the abundance data, we superimposed the $M(t = endpoint)$ prediction onto the observed abundance distribution from sequencing data (blue (f=1), red (f=0), purple (f=0.5) vertical lines in Extended Data Fig. 7i) and calculated a one-tailed p-value based on the quantile the prediction lies in the data distribution in each phylum and pH combination. In pH 5, 6, and 7 for all phyla, we saw no significant deviation of observed abundance distribution from the no-death model predictions (p-values ranging from 0.1 to 0.4). This result also provides significant evidence that chloramphenicol does not cause cell death, allowing us to rule out chloramphenicol-induced death across pH conditions. Furthermore, Bacillota showed no significant death even in pH 3 (when f=0.5, p=0.73; when f=1, p=0.13), pH 3.5 (when f=0.5, p=0.81; when f=1, p=0.38), and pH 4 (when f=0.5, p=0.65; when f=1, p=0.46).

During acidic perturbations of Regime I (pH 3, 3.5, and 4), we observed strong evidence for widespread death in phyla Pseudomonadota and Bacteroidota. For pH 3.5 and 4, the distribution of endpoint abundance data deviated significantly from the no-death model predictions in all scenarios ($p < 0.05$ in f=0, f=0.5, f=1) (Extended Data Fig. 7j). Even in extremely acidic pH 3 conditions, where the DNA degradation rate is very high, the abundance data cannot be explained without acid-induced death (when f= 0.5, p < 0.002 for Pseudomonadota and p < 0.011 for Bacteroidota). Given the constant rates of DNA degradation irrespective of the phylum, we regard this as a strong case for death at least partially accounting for the abundances we observe.

Inferring the fraction of relic DNA with the model and abundance data

To estimate the fraction of relic DNA (f) from our data, we can go one step further with our model (equations (5)) using both the death rate d and DNA degradation rate δ measured from our experiments. Estimating f can provide insight into how much of the signal in sequencing data is coming

from the actual microbial biomass. We used the family abundance data from the Pseudomonadota phylum in chloramphenicol-treated slurry samples where endpoint pH levels were 3.5, 4, or 5 ($\pm$0.2), assuming that f is not dependent on the perturbed pH levels but dependent on the starting soil. For d and δ values, we used the DNA degradation and death rate measurements in pH 3.5, 4, and 5 conditions from *Massilia* strain to represent Pseudomonadota. To fit the f parameter with the data points for each family, soil, and pH combination, we set up the loss function as the root-mean-squared-error (RMSE) between the normalized endpoint abundance ($M(t = endpoint)/M(0)$) predicted by the model and the observed data. Because the fitting of f is sensitive to the α parameter, we fixed the α at varying levels (from 0 to 1 in intervals of 0.1) and performed fitting f with the L-BFGS-B algorithm. There was substantial variation in predicted f depending on the soil and α even for fits having RMSE < 0.2. This could be due to the varying proportion of relic DNA depending on the family. To summarize the f estimates for each soil, we computed the median f from the best f fits (minimum RMSE) across varying fixed α values for each combination of soil, family, and pH condition, as multiple α values often resulted in minimum RMSE fits. The median fraction of relic DNA in different soils was estimated to range from 0 to approximately 0.4.

Justifying the effective 1-biomass model despite the diversity of denitrifying taxa

The paper uses a model where the "functional biomass" is assumed to be represented by a single group. In reality, nitrate utilization is performed by multiple taxa, which will in general, have distinct uptake rates, nutrient affinities, and growth rates. One might be concerned that this fact might undermine the utility of the 1-biomass model for describing nitrate dynamics. In this section, we use simulations to demonstrate the kinds of deviations one expects to observe and the parameter regime where these deviations would be largest. We will then show a supporting example in our data. Nevertheless, our analysis will demonstrate that at the precision of the experiment, the 1-biomass model is expected to provide a very good approximation to nitrate dynamics even when many taxa are involved.

To investigate this question, consider the following model with K denitrifying taxa:

$$\begin{cases} \dot{A}(t) = -\sum_{i=1}^{K} r_{Ai}\, x_i(t)\, \frac{A(t)}{A(t)+K_{Ai}} \\ \dot{C}(t) = -\sum_{i=1}^{K} r_{Ci}\, x_i(t)\, \frac{C(t)}{C(t)+K_{Ci}} \\ \dot{x}_i(t) = \gamma_i\, x_i(t)\, \frac{A(t)}{A(t)+K_{Ai}} \frac{C(t)}{C(t)+K_{Ci}}. \end{cases} \tag{9}$$

For simplicity, we first consider the case where carbon limitation is not a major factor (i.e., nitrate runs out before carbon, as in Regime III in the main text). In this scenario, the key parameters of each species are the initial abundance $x_i(0)$ and the growth rate γ_i (we set the affinity K_{Ai} to be low for all i, as in the main text). For our illustration, we will randomly generate sets of $K = 10$ species, drawing these parameters out of a heavy-tailed distribution of varying widths σ_x and σ_γ:

$$\log(x) \sim \mathcal{N}(0.01, \sigma_x) \text{ and } \log(\gamma) \sim \mathcal{N}(0.005, \sigma_\gamma)$$

For each of these 10-species sets, we will simulate the "true" 10-group dynamics according to Eqs. (9) up until nitrate is fully depleted, and record also the "CHL^+" version of the dynamics ("with chloramphenicol", setting $\gamma = 0$ for all species). We then fit these nitrate utilization curves to a 1-biomass model (described by the same equations with $K = 1$; this fitting is performed exactly as in the main text, jointly for the CHL^+ and CHL^- curves). The root-mean-squared (RMS) error of this fit characterizes the ability of this effective 1-biomass description to approximate the 10-group dynamics. Note that in order to highlight the differences between the models, this procedure ignores any experimental noise, which in practice would contribute to masking any discrepancies.

Extended Data Fig. 9a shows the average RMS error of such a fit, as a function of the width of the distributions of the initial abundances x and growth rates γ of the species. Note that the widths are in log10 space, so a value of 1 corresponds to a very wide distribution (68% of values are contained in the interval from 10-fold below typical to 10-fold over typical). Each pixel represents an average over 100 random realizations.

As expected, if all species have similar growth rates, they behave as one (the lower half of the heatmap is all blue; low RMS error). If the initial abundances are strongly unequal, nitrate consumption will typically be dominated by a single (most abundant) species, so the 1-biomass model again performs well. The largest error is observed when species have comparable initial abundance, but vary strongly in growth rate (upper left corner; highest RMS error). An example of behavior with such parameters is shown in panel B. Inspecting this example reveals the failure mode of the 1-biomass model: this effective model can exhibit either a significant late-time growth, or a large initial utilization slope, but not both. Indeed, if we are limited to a single taxon, observing an exponential late-time growth *requires* that taxon to start from a very low initial abundance.

This indicates for us the regime in which the 1-biomass model is expected to struggle the most: namely, combining slow growers with a large $x(0)$ with fast growers that start from small $x(0)$. This scenario can exhibit both an initial slope (contributed by the former) and a late-time growth (contributed by the latter). To see this, we repeated the same analysis, but enforced a perfect anti-correlation between x_0 and γ (slow growers are present at larger abundance, and fast growers are present at low abundance). The results are shown in Extended Data Fig. 9c, and confirm that the fit quality decreases as expected (RMS error is larger). Panel D shows an example from the large-error corner ($\sigma_x = 0.1$, $\sigma_\gamma = 0$). As expected, fitting the accelerated late-time depletion causes the 1-biomass model to underfit the initial slope. We note, however, that even in this "worst-case scenario", the deviations are comparable to the experimental noise.

This analysis tells us that the largest deviations from the 1-biomass fit are expected to occur during the transition from Regime II to Regime III ("resurgent growth"). Indeed, as we describe, in Regime II denitrification is dominated by initially abundant species with moderate additional growth, whereas in Regime III we observe explosive growth of initially negligible Bacillota. The boundary between these two regimes is where one might expect both groups to contribute, setting up the conditions for the 1-biomass model to fail.

Examining the transition regime, we do indeed observe the deviations of the kind illustrated

in Fig. 9c. The strongest example is in soil 16, and Extended Data Fig. 9e shows the three most basic perturbations for this soil. While the extreme basic condition is consistent with the explosive growth of a single group, both boundary cases are indicative of a late-time growth of the kind that is incompatible with the 1-biomass effective model, and this signature is consistent across the three replicates (shown separately for clarity). However, this effect is very subtle, and even in this regime, the 1-biomass model correctly captures the fact that the late-time growth must indicate a large metabolic activity of a taxon that starts at low initial abundance—the signature of the "Resurgent growth" regime, independently confirmed with the sequencing data.

For simplicity, the discussion above focused on the case where carbon limitation was not a major factor (nitrate runs out first). To close this section, we comment on the effect of carbon limitation, including a scenario where different taxa are limited by different carbon sources. Note that once the growth of *all* taxa is arrested by carbon exhaustion, the nitrate uptake rate ceases to increase, and the utilization curve becomes linear. Such linear dynamics are fully compatible with the single-biomass effective model. Therefore, any differences between the two models can be observed only during the transient (while the limiting nutrient(s) have not yet been fully consumed).

Empirically, in most conditions of Regime II, the initial growth burst is completed within the time span covered by the first 2-3 data points. Because of this, the dynamical differences that might have required the more complex model simply cannot be resolved. The only scenario not covered by this argument is a situation where different species run out of their carbon at significantly different times, resolvable at the temporal resolution of the experimental sampling. This scenario can indeed generate utilization profiles that differ significantly from those achievable by a single-biomass model, showing multiple distinct growth bursts. Extended Data Fig. 9f shows an example that would be most distinguishable with our measurements (two significant growth bursts, one occurring very early, the other very late). Extended Data Fig. 9g shows the closest example in our data. The high accuracy of the measurement makes it quite plausible that the trace shown constitutes a genuine example of an early growth burst of one taxon, followed by a late-time growth

of a taxon limited by a different nutrient. However, even in this case, the 1-biomass model provides an excellent fit (see Extended Data Fig. 9h), justifying our use of this effective model.

Prediction of nitrate dynamics from community structure

Our model enables *predictions* of function (nitrate utilization dynamics) directly from sequencing and metabolite measurements. The two key model parameters $C(0)$ and $x(0)$ can be estimated using measurements of water-soluble organic carbon (WSOC) and the sequencing data, respectively. Subsequently, given the fixed value of model parameters inferred from training data ($K_A = 0.01mM$, $\tilde{K}_C = 0.01mM$, $\gamma = 0.48day^{-1}$), we can simulate the model and predict the nitrate utilization dynamics for microcosms not used during model fitting. The approach is as follows: for a training subset of the data (soils and pH perturbations).

- (Step 1) Find a sequencing-based quantity that correlates with $x(0)$.
- (Step 2) Find a WSOC measurement that correlates with $C(0)$.
- (Step 3) Use $\tilde{x}(0)$, $\tilde{C}(0)$ inferred from nitrate dynamics and the results of step 1 to infer $r_A = \tilde{x}(0)/x(0)$ and $r_A/r_C = \tilde{C}(0)/C(0)$. Try two models for r_A and r_A/r_C as a function of pH:
  - (Model 1) Constant model: both quantities are constants, inferred as the mean across all samples.
  - (Model 2) Use non-parametric regression to infer the function $r_A(pH) = f_1(pH)$ and $r_A/r_C(pH) = f_2(pH)$
- (Step 4) For the test data:
  - (4.1) Predict r_A and r_A/r_C (via either Model 1 or Model 2).
  - (4.2) Use predictions for $x(0)$ and $C(0)$ from Steps 1 and 2 and the predictions from 4.1 to predict $\tilde{x}(0) = r_A x(0)$ and $\tilde{C}(0) = r_A/r_C C(0)$.

– Integrate the model (Fig. 2) using $\tilde{x}(0)$ and $\tilde{C}(0)$ (Methods) to predict $A(t_k)$ and compare to measured a_k.

(Step 1) The first step is to estimate $x(0)$ using an appropriate quantity in the sequencing. To achieve a good estimation of $x(0)$, the sequencing quantity should *correlate* with the $\tilde{x}(0)$ inferred from nitrate dynamics (Extended Data Fig. 10a). We therefore examined the Pearson's correlation coefficient $\rho = E[X(0)\tilde{X}(0)]/\sqrt{E[X(0)^2]E[\tilde{X}(0)^2]}$ and the variance of log-ratio $\sigma^2 = Var[lg(\tilde{X}(0)/X(0))]$. We found that the inferred abundance of nitrate reductases had the highest ρ and smallest σ^2, indicating that the abundance of functional genes had the highest correlation with $\tilde{x}(0)$. We thus took the functional gene abundance as the estimated $x(0)$. The functional gene abundance is the summed abundance of all ASVs that possess Nar and Nap genes in CHL+ samples inferred via PICRUST2 [28].

(Step 2) Similarly, for $C(0)$ estimation, we computed the correlations ρ and σ^2 between the different measurements of carbon in the system (Extended Data Fig. 10b) quantity and inferred $\tilde{C}(0)$ among the WSOC measurements. We found that WSOC measurements at the endpoint of the experiment in CHL- samples had the highest correlation with $\tilde{C}(0)$, and thus we chose this quantity as the estimated $C(0)$ (Extended Data Fig. 10b).

(Step 3) Given the good estimations of $x(0)$ and $C(0)$, the next necessary step was to set two model parameters r_A and r_C to predict $\tilde{x}(0) = x(0)r_A$ and $\tilde{C}(0) = C(0)r_A/r_C$. To do so, we separated the whole dataset (10 soils) into training and test datasets, each containing the data of 5 soils. We then used the nitrate dynamics (yielding $\tilde{x}(0), \tilde{C}(0)$), sequencing, and WSOC measurements in the training dataset to learn r_A and r_C with the given features of the two parameters. After the learning step, we can use the learned r_A and r_C, together with the estimations of $C(0)$ and $x(0)$, to simulate and predict the nitrate dynamics in the test dataset. There are two ways the model can learn r_A and r_C with different features. (Model 1) In the first model, we assume r_A and r_C are constant parameters across all conditions. Since $r_A = \tilde{x}(0)/x(0)$, we used the mean value of $lg(\tilde{x}(0)/x(0))$ as the learned $lg(r_A)$ and took the range of 0.5 standard deviations as the learning

uncertainty on the parameter (Extended Data Fig. 10c). Since $r_A/r_C = \tilde{C}(0)/C(0)$, we used the mean value of $lg(\tilde{C}(0)/C(0))$ as the learned $lg(r_A/r_C)$ and took the range of 0.5 standard deviations as the learning uncertainty on the parameter (Extended Data Fig. 10c). However, r_A and r_C do not necessarily need to be constants. (Model 2) Therefore, in the second model, we assume r_A and r_C are functions of pH using the pH values and $lg(\tilde{x}(0)/x(0))$ values in the training dataset to learn the pH-dependent function $lg(r_A) = f_1(pH)$. We used the pH values and $lg(\tilde{C}(0)/C(0))$ values in the training dataset to learn the pH-dependent function $lg(r_A/r_C) = f_2(pH)$. Gaussian kernel smoother $K(pH, pH_i) = \exp(-(pH - pH_i)^2/2s^2)$ were used to learn smooth function curves:

$$\hat{y}(pH) = \frac{\sum_i K(pH, pH_i) y_i}{\sum_i K(pH, pH_i)}, \tag{10}$$

where y_i is the i-th data of $lg(\tilde{x}(0)/x(0))$ or $lg(\tilde{C}(0)/C(0))$. The kernel width $s = 0.25$ is chosen to include one sample per soil within 1 standard deviation of the kernel. We took $\hat{y}(pH)$ as the learned f_1 or f_2 function and the range of 0.5 standard deviations of $\hat{y}$ as the learning uncertainty on the function (Extended Data Fig. 10d).

(Step 4) Since now we estimated $x(0)$, $C(0)$ and learned r_A, r_C on the training data from 5 soils we can predict $\tilde{x}(0)$ and $\tilde{C}(0)$ and predict nitrate dynamics for the 5 soils that were not used in training. Extended Data Fig. 10f shows three examples of nitrate data and the predictions, where one example nitrate time series is chosen from each Regime. We found that the pH-dependent model (Model 2) predicts well in all three Regimes. Across all samples in the training dataset, the root-mean-squared-error (RMSE) was only $0.20mM$, while the prediction error was $RMSE = 0.28mM$ in the test dataset (Extended Data Fig. 10g). Model 1 (constant r_A and r_A/r_C) predicted Regime II and III dynamics well but failed in Regime I. This suggests that r_A and r_C are altered at low pH levels.

Lastly, to assess the predictive power purely from pH, we investigated a pH-null model, by which $\tilde{x}(0)$ and $\tilde{C}(0)$ directly learned as two functions of pH (Extended Data Fig. 10e). In the pH-null model, structures (carbon and sequencing abundance) were neglected, and only the pure environment-function mapping was established. The prediction results presented in Extended Data

Fig. 10f indicate that three Regimes can be learned from environment-function mapping. However, it failed in quantitative predictions of nitrate reduction rate, especially in Regime II. We conclude that the scheme outlined above reliably maps sequencing and carbon measurements to nitrate dynamics.

In summary, our model gives accurate predictions of nitrate utilization dynamics. We used the functional gene abundances in CHL+ sample and WSOC measurements in CHL- sample at the endpoint to estimate $x(0)$ and $C(0)$ in the model. Then, we learned the parameters r_A and r_C as pH-dependent functions. Model simulations for nitrate dynamics successfully captured the dynamic features corresponding to each Regime and accurately predicted the observed nitrate consumption dynamics.

Supplementary Tables

Table S1: Soil sample information and physicochemical properties

Soil No.	Soil ID	pH_{H2O}	Latitude	Longitude	Sand (%)	Silt (%)	Clay (%)	C (mg/g)	N (mg/g)	C:N ratio	Depth	Sampling (M/D/YY)
Soil1	Acidic4	4.703	46.785703	-117.079245	0.00	58.15	41.85	16.12	1.26	12.79	0-10cm	9/10/22
Soil2	Acidic12	5.094	46.781117	-117.080513	0.00	63.80	36.20	19.66	1.49	13.19	0-10cm	9/12/22
Soil3	CE239	4.987	46.781153	-117.080455	0.00	61.20	38.80	13.50	1.10	12.27	10-20cm	9/8/22
Soil4	SE56b	5.277	46.778744	-117.082738	0.00	57.40	42.60	12.64	0.95	13.31	10-20cm	9/8/22
Soil5	CE201	5.324	46.781049	-117.086242	0.00	58.70	41.30	15.68	1.20	13.07	10-20cm	9/8/22
Soil6	CE73	5.405	46.779637	-117.086172	0.00	61.20	38.80	16.47	1.27	12.97	10-20cm	9/8/22
Soil7	CE153	5.514	46.7805	-117.085431	0.00	58.70	41.30	17.55	1.27	13.82	10-20cm	9/8/22
Soil8	CE56a	5.552	46.778855	-117.082968	0.00	58.70	41.30	13.50	0.99	13.64	10-20cm	9/8/22
Soil9	CE277	5.822	46.781883	-117.0835	0.00	61.20	38.80	11.89	0.94	12.65	10-20cm	9/8/22
Soil10	CE253	5.975	46.781534	-117.084192	0.00	62.50	37.50	9.84	0.82	12.00	10-20cm	9/8/22
Soil11	CE234	6.186	46.781301	-117.082533	0.00	62.50	37.50	18.81	1.34	14.04	10-20cm	9/8/22
Soil12	CE229	6.255	46.781206	-117.084623	0.00	58.80	41.20	11.97	0.94	12.73	10-20cm	9/8/22
Soil13	Neutral7	6.435	46.781523	-117.084533	0.00	60.00	40.00	9.89	0.82	12.06	10-20cm	9/11/22
Soil14	Neutral2	6.545	46.781308	-117.084696	0.00	58.80	41.20	12.78	1.06	12.06	10-20cm	9/11/22
Soil15	Neutral5	6.789	46.781416	-117.084818	0.00	63.70	36.30	13.10	0.95	13.79	10-20cm	9/11/22
Soil16	Neutral6	6.860	46.781524	-117.084694	0.00	61.20	38.80	10.70	0.87	12.30	10-20cm	9/11/22
Soil17	Neutral3	7.052	46.781194	-117.084732	0.00	60.00	40.00	10.93	0.87	12.56	10-20cm	9/11/22
Soil18	Neutral1	7.681	46.781354	-117.084812	0.00	63.70	36.30	12.12	0.98	12.37	10-20cm	9/11/22
Soil19	Neutral4	8.232	46.781222	-117.084882	0.00	62.50	37.50	10.47	0.76	13.78	10-20cm	9/11/22
Soil20	CE251	8.323	46.781492	-117.085028	0.00	67.50	32.50	14.69	0.91	16.14	10-20cm	9/8/22
LaBagh	IL	6.36	41.977805	-87.742510	60.00	27.50	12.50	27.30	2.10	13.00	0-10cm	8/26/24
Pinhook	IN	5.76	41.621229	-86.850022	27.50	52.50	20.00	25.20	2.14	11.78	0-10cm	8/27/24
CLG13	Sedgwick	6.27	34.694032	-120.047434	27.50	45.00	27.50	24.20	2.48	9.76	0-10cm	8/20/24
ELG13	Sedgwick	6.38	34.716462	-120.060468	41.30	35.00	23.70	27.25	2.30	11.85	0-10cm	8/22/24

Supplementary references

[71] Bergaya, F. & Lagaly, G. *General introduction: clays, clay minerals, and clay science*, vol. 5, 1–19 (Elsevier, 2013).

[72] Price, M. N., Dehal, P. S. & Arkin, A. P. Fasttree 2–approximately maximum-likelihood trees for large alignments. *PLoS One* **5**, e9490 (2010).

[73] Galperin, M. Y. Genome diversity of spore-forming firmicutes. *Microbiol. Spectr.* 1–18 (2013).

[74] An, R. *et al.* Non-Enzymatic Depurination of Nucleic Acids: Factors and Mechanisms. *PLoS One* **9**, e115950 (2014).

[75] Heylen, K. *et al.* Cultivation of Denitrifying Bacteria: Optimization of Isolation Conditions and Diversity Study. *Appl. Environ. Microbiol.* **72**, 2637–2643 (2006).

[76] Schindelin, J. *et al.* Fiji: an open-source platform for biological-image analysis. *Nature Methods* **9**, 676–682 (2012).

Meta-analysis of electron acceptor dynamics during denitrification in soil microcosms

Abstract

Background: Denitrification by bacteria in soils is critical for nitrogen cycling and greenhouse gas emissions. However, variations in the soil types and experimental conditions across studies have led to differing conclusions about how microbial denitrification is influenced by environmental conditions. pH and carbon availability are the chief environmental correlates with denitrification rates.

Objectives: The objective is to test the hypothesis that functional regimes described by differing rates and dynamics of nitrate utilization across pH conditions are evident in other studies using soil microcosms. Functional regimes are characterized by the values of two parameters for a model of community metabolism: indigenous biomass activity and available limiting nutrients.

Methods: Due to the breadth of denitrification literature, and the fact that including a study in our metanalysis required dynamic measurements of either abundances or metabolites, we were unable to use general search terms to define a reasonably sized pool of potential studies. Instead, we performed a search based on forward/backward citation searches from an inclusive library of the key papers in the field. Inclusion criteria required presence of quantitative measurements of denitrification rates or metabolite dynamics, soil pH, and metadata (C/N ratio, soil particle composition). The risk of bias was assessed via how well soils spanned the soil texture (particle composition) classification triangle (**Fig. 6A**).

Results: Our meta-analysis confirmed that distinct functional regimes (Regime II and III) exist in diverse soils. Across soils with different native pH levels, the transition from Regime II to Regime III during basic perturbations was observed (**Fig. 6C**) In addition, Denitrification enzyme activity declined under strong pH perturbations across multiple independent datasets, supporting the pattern of reduced biomass activity in Regimes I and III (**Fig. 6F**). Nitrogen utilization dynamics revealed both the linearity near native pH and increase in reduction rates upon carbon amendment, mirroring the behavior predicted in Regime II (**Fig. 6G**).

Discussion: Our re-analysis of historical datasets revealed consistent functional regimes despite differences in soil type and experimental design, supporting the generality of our framework. The separation of early and late denitrification rates allowed us to infer the two parameters of our model. These findings demonstrate that functional regimes during pH perturbation are robust and observable in legacy datasets.

Funding: This work was supported by the National Science Foundation Division of Emerging Frontiers EF 2025293 (S.K.) and EF 2025521 (M.M.) and by National Science Foundation PHY 2310746 (M.T.). S.K. acknowledges the National Institute of General Medical Sciences R01GM151538, and support from the National Science Foundation through the Center for Living Systems (grant no. 2317138). S.K. and M.T. acknowledge CAREER awards from the

National Science Foundation (BIO/MCB 2340416 and PHY-2340791). S.K. and M.M. acknowledge financial support from the National Institute for Mathematics and Theory in Biology (Simons Foundation award MP-TMPS-00005320 and National Science Foundation award DMS-2235451). MM was supported by The National Science Foundation-Simons Center for Quantitative Biology at Northwestern University and the Simons Foundation grant 597491. MM is a Simons Investigator. This project has been made possible in part by grant number DAF2023-329587 from the Chan Zuckerberg Initiative DAF, an advised fund of the Silicon Valley Community Foundation. Any opinions, findings, conclusions, or recommendations expressed in this material are those of the authors and do not necessarily reflect the views of the National Science Foundation.

Introduction

Rationale: While many studies have examined denitrification in soils, differences in soil types and experimental designs have led to varying interpretations of how microbial communities utilize nitrate in response to changing environmental conditions like pH and nutrient availability. Revisiting these studies through a common mechanistic lens may reveal consistent patterns that were not previously apparent.

Objectives: This meta-analysis aims to assess whether the functional regimes identified in our framework are also present in historical denitrification studies. Rather than exhaustively surveying the literature, we focus on re-analyzing representative time-series datasets to validate the robustness and generality of our proposed mechanistic regimes.

Methods

Eligibility criteria: We included studies that reported quantitative measurements of denitrification rates or nitrogen compound dynamics over time, along with metadata on pH, carbon content/amendment, and soil properties. We focused on studies with time-resolved data sufficient to evaluate functional regimes.

Information sources: We manually searched peer-reviewed articles using Google scholar and Web of Science.

Search strategy: No formal database filters or automation tools were used. Because the inclusion criteria were so stringent, we could not survey all denitrification-related literature. Instead we took a forward/backward citation search approach. We identified several key papers in the field and began searching every paper that was cited, or cited that paper. We seeded this search with three key papers. For any papers cited or that cited one of these papers, we examined the abstract and figures. Any papers with usable data then seeded the next round of search via citations and cited articles. This yielded approximately 15 papers that were considered in detail, 9 of which we were able to quantitatively analyze the available data. Approximately 200 papers were considered during this search.

Selection process: Studies were selected based on whether plots showed measurable denitrification dynamics (e.g., nitrate, nitrite, N_2O, N_2) with corresponding metadata. Authors

(S.K., K.K.L., S.L.) screened and selected the studies, focusing on diversity in soil type, geographic location, and experimental design.

Data collection process: Data were extracted manually from published figures via ImageJ. One author (S.L.) handled the digitization and metadata annotation for all studies. No contact with study authors was made for raw data retrieval.

Data items:
10a. Primary outcomes were denitrification enzyme activity (DEA), denitrification potential (DP), and time-resolved concentrations of nitrogen compounds, either nitrate or nitrous oxide. We used early- and late-stage rates for inferring microbial activity and nutrient limitation via our model.

10b. Additional variables included soil texture, native pH, treatment pH, carbon amendments, and C/N ratio. When metadata were missing, we inferred approximate values based on descriptions in the methods sections or accompanying tables. For example, if clay:silt:sand ratios were not published, but a soil type name was published, we took median values for the clay:silt:sand ratios for that soil type.

Study risk of bias assessment: We minimized the risk of missing potential studies via the repeated seeding of our branching search process. We assessed the risk that our scientific findings were biased by the representativeness of the soil types (triangular soil texture classification in **Fig. 6A**).

Effect measures:
We did not compute traditional effect sizes. Instead, we used slopes of early and late denitrification phases to infer microbial parameters (e.g., $\tilde{x}(0)$, $\gamma\tilde{C}(0)$) and tracked directional changes across pH and carbon perturbation gradients. There was no statistical test performed.

Synthesis methods:
13a. We selected studies that had sufficient temporal resolution and metadata to allow inference of functional parameters and mapping to our regime framework.
13b. When necessary, we converted visual data into numerical form by digitizing plots. Early linear fits were used for $\tilde{x}(0)$; the maximum slope of non-linear curves was used for $\gamma\tilde{C}(0)$.
13c. Results were displayed using scatter plots (e.g., **Fig. 6C, F, G**), and additional metadata were compiled in **Table S2**.
13d. No formal statistical meta-analysis was conducted. We used qualitative synthesis of rate comparisons across regimes to assess consistency with our theoretical predictions. As claimed in the manuscript, the conservation of regimes is a qualitative result.
13e. Soil type, native pH, and treatment conditions were used to explain heterogeneity in observed patterns.
13f. Sensitivity was checked by comparing patterns across subsets of studies, and no qualitative differences were observed.

Reporting bias assessment: We acknowledge a likely reporting bias toward studies that measured denitrification dynamics or had sufficient perturbation conditions.

Certainty assessment: Certainty in our findings was evaluated based on the consistency of observed patterns across independent studies and parameter inference across various soil types. Qualitative agreement across studies increases confidence in the generality of the regimes.

Results

Study Selection

16a. Our meta-analysis identified 9 representative denitrification studies spanning 70 years (1956-2024) that met our inclusion criteria. These studies provided 19 soil samples with time-series measurements of denitrification dynamics under pH-perturbed or carbon-amended conditions.

16b. Several studies that appeared relevant were excluded because they lacked either (1) quantitative time-series measurements of denitrification-related metabolites, (2) sufficient metadata about soil properties (pH, C/N ratio, particle composition), or (3) clear experimental conditions for pH or carbon perturbations. These exclusions ensured only studies with interpretable dynamics and complete metadata were analyzed.

Study Characteristics:

The included studies represented 19 diverse soils:

- Anderson et al. (2018): 1 New Zealand soil (agriculture)
- Šimek et al. (2002): 5 Czech soils (arable land, grassland)
- Parkin et al. (1985): 2 Michigan soils (agriculture)
- Nömmik (1956): 2 Norwegian soils (unknown land use)
- Bremner and Shaw (1958a): 2 UK soils (arable)
- Bremner and Shaw (1958b): 2 UK soils (pasture)
- Šimek & Hopkins (1999): 1 Czech soil (arable)
- Khalifa & Folz (2024): 4 Oklahoma soils (grassland)

These covered silty clay loams, sandy loams, and clay soils across Europe and North America (**Fig. 6A**, **Table S2**).

Risk of Bias: The risk of bias was assessed based on soil representativeness (covering the soil texture classification triangle) and completeness of metadata (pH, C/N, particle composition). All included studies may contain bias. Studies occasionally missed C/N data, land usage or approximate geolocations.

Results of Individual Studies:

- Anderson et al. (2018): Confirmed enrichment of Bacillota in basic perturbations (**Fig. 6E**)
- Šimek et al. (2002): DEA/DP measurements evidencing Regime I, II, and III (**Fig. 6C**)
- Parkin et al. (1985): Indigenous biomass activity decreases in extreme perturbations both in acid/neutral soil (**Fig. 6F**)
- Nömmik (1956): Linear dynamics found in Regime II (**Fig. 6G** right)
- Bremner and Shaw (1958): Linear dynamics found in Regime II and transition to Regime III due to carbon amendments (**Fig. 6G** left)

- Šimek & Hopkins (1999): Indigenous biomass activity decreases in extreme perturbations both in acid/neutral soil (**Fig. 6F**)
- Khalifa & Folz (2024): Indigenous biomass activity decreases in extreme perturbations both in acid/neutral soil (**Fig. 6F**)

Synthesis Results:
20a. The nine studies consistently showed (1) Regime II (linear dynamics near native pH) and (2) Regime III during basic pH perturbations. Fewer studies showed Regime I due to a lack of data without antibiotics and acidic pH perturbations.
20b. We did not statistically synthesize the denitrification data from different soils. Instead, we show for individual soils the directional changes across pH and carbon perturbation gradients (**Fig. 6**).
20cd. Soil type and different experimental conditions may have influenced the denitrification dynamics. However, the qualitative behavior of distinct soils reflected functional regimes.

Reporting Biases: Potential biases could be the overrepresentation of European soils and the exclusion of studies without time-series data and limited tropical/arid soil coverage.

Certainty of Evidence: High certainty exists for functional Regime II and Regime III and carbon limitation in Regime II. Limited evidence in these studies is available for Regime I since only one study showed this regime. Limitations include methodological heterogeneity and small sample size (soil n=19).

Discussion

23a. The consistent observation of functional regimes across diverse soils and experimental conditions suggests these regimes are not only confined to Cook Agronomy Farm soils but are generalizable to other soils.
23b. While consistent within the analyzed dataset, the evidence in literature remains limited by the handful number of dynamic measurements available, heterogeneous methodologies across studies, and underrepresentation of certain soil types (e.g., tropical, arid).
23c. Our review process prioritized studies with time-series data, potentially excluding relevant static measurements, and relied on digitized plots where raw data were unavailable.
23d. These findings highlight the importance of making dynamic measurements in future denitrification studies.

Other information

Registration and Protocol: This meta-analysis was not formally registered other than being part of the manuscript, and no protocol was prepared prior to analysis.

Support: Funding was provided by NSF, NIH, Simons Foundation, and CZI grants; all funders had no role in study design or interpretation.

Competing Interests: The authors declare no financial conflicts.

Availability of Materials: Metadata table (**Table S2**) is provided in supplementary files. The data and analysis code are deposited at the [Open Science Framework](https://osf.io/ctf8k/) (https://osf.io/ctf8k/).

Section	Item #	Checklist item	Location where item is reported
**TITLE**			
Title	1	Identify the report as a systematic review.	Page 1
**ABSTRACT**			
Abstract	2	See the PRISMA 2020 for Abstracts checklist.	Page 1
**INTRODUCTION**			
Rationale	3	Describe the rationale for the review in the context of existing knowledge.	Page 2
Objectives	4	Provide an explicit statement of the objective(s) or question(s) the review addresses.	Page 2
**METHODS**			
Eligibility criteria	5	Specify the inclusion and exclusion criteria for the review and how studies were grouped for the syntheses.	Page 2
Information sources	6	Specify all databases, registers, websites, organisations, reference lists and other sources searched or consulted to identify studies. Specify the date when each source was last searched or consulted.	Page 2
Search strategy	7	Present the full search strategies for all databases, registers and websites, including any filters and limits used.	Page 2
Selection process	8	Specify the methods used to decide whether a study met the inclusion criteria of the review, including how many reviewers screened each record and each report retrieved, whether they worked independently, and if applicable, details of automation tools used in the process.	Page 2
Data collection process	9	Specify the methods used to collect data from reports, including how many reviewers collected data from each report, whether they worked independently, any processes for obtaining or confirming data from study investigators, and if applicable, details of automation tools used in the process.	Page 3
Data items	10a	List and define all outcomes for which data were sought. Specify whether all results that were compatible with each outcome domain in each study were sought (e.g. for all measures, time points, analyses), and if not, the methods used to decide which results to collect.	Page 3
	10b	List and define all other variables for which data were sought (e.g. participant and intervention characteristics, funding sources). Describe any assumptions made about any missing or unclear information.	Page 3
Study risk of bias assessment	11	Specify the methods used to assess risk of bias in the included studies, including details of the tool(s) used, how many reviewers assessed each study and whether they worked independently, and if applicable, details of automation tools used in the process.	Page 3
Effect measures	12	Specify for each outcome the effect measure(s) (e.g. risk ratio, mean difference) used in the synthesis or presentation of results.	Page 3
Synthesis methods	13a	Describe the processes used to decide which studies were eligible for each synthesis (e.g. tabulating the study intervention characteristics and comparing against the planned groups for each synthesis (item #5)).	Page 3
	13b	Describe any methods required to prepare the data for presentation or synthesis, such as handling of missing summary statistics, or data conversions.	Page 3
	13c	Describe any methods used to tabulate or visually display results of individual studies and syntheses.	Page 3
	13d	Describe any methods used to synthesize results and provide a rationale for the choice(s). If meta-analysis was performed, describe the model(s), method(s) to identify the presence and extent of statistical heterogeneity, and software package(s) used.	Page 3
	13e	Describe any methods used to explore possible causes of heterogeneity among study results (e.g. subgroup analysis, meta-regression).	Page 3
	13f	Describe any sensitivity analyses conducted to assess robustness of the	Page 3

Section	Item #	Checklist item	Location where item is reported
		synthesized results.	
Reporting bias assessment	14	Describe any methods used to assess risk of bias due to missing results in a synthesis (arising from reporting biases).	Page 3
Certainty assessment	15	Describe any methods used to assess certainty (or confidence) in the body of evidence for an outcome.	Page 4
**RESULTS**			
Study selection	16a	Describe the results of the search and selection process, from the number of records identified in the search to the number of studies included in the review, ideally using a flow diagram.	Page 4
	16b	Cite studies that might appear to meet the inclusion criteria, but which were excluded, and explain why they were excluded.	Page 4
Study characteristics	17	Cite each included study and present its characteristics.	Page 4
Risk of bias in studies	18	Present assessments of risk of bias for each included study.	Page 4
Results of individual studies	19	For all outcomes, present, for each study: (a) summary statistics for each group (where appropriate) and (b) an effect estimate and its precision (e.g. confidence/credible interval), ideally using structured tables or plots.	Page 4
Results of syntheses	20a	For each synthesis, briefly summarise the characteristics and risk of bias among contributing studies.	Page 5
	20b	Present results of all statistical syntheses conducted. If meta-analysis was done, present for each the summary estimate and its precision (e.g. confidence/credible interval) and measures of statistical heterogeneity. If comparing groups, describe the direction of the effect.	Page 5
	20c	Present results of all investigations of possible causes of heterogeneity among study results.	Page 5
	20d	Present results of all sensitivity analyses conducted to assess the robustness of the synthesized results.	Page 5
Reporting biases	21	Present assessments of risk of bias due to missing results (arising from reporting biases) for each synthesis assessed.	Page 5
Certainty of evidence	22	Present assessments of certainty (or confidence) in the body of evidence for each outcome assessed.	Page 5
**DISCUSSION**			
Discussion	23a	Provide a general interpretation of the results in the context of other evidence.	Page 5
	23b	Discuss any limitations of the evidence included in the review.	Page 5
	23c	Discuss any limitations of the review processes used.	Page 5
	23d	Discuss implications of the results for practice, policy, and future research.	Page 5
**OTHER INFORMATION**			
Registration and protocol	24a	Provide registration information for the review, including register name and registration number, or state that the review was not registered.	Page 5
	24b	Indicate where the review protocol can be accessed, or state that a protocol was not prepared.	Page 5
	24c	Describe and explain any amendments to information provided at registration or in the protocol.	Page 5
Support	25	Describe sources of financial or non-financial support for the review, and the role of the funders or sponsors in the review.	Page 5
Competing interests	26	Declare any competing interests of review authors.	Page 5
Availability of data, code and other materials	27	Report which of the following are publicly available and where they can be found: template data collection forms; data extracted from included studies; data used for all analyses; analytic code; any other materials used in the	Page 5

Section	Item #	Checklist item	Location where item is reported
		review.	

Abstract checklist

Section and Topic	Item #	Checklist item	Reported (Yes/No)
**TITLE**			
Title	1	Identify the report as a systematic review.	Yes
**BACKGROUND**			
Objectives	2	Provide an explicit statement of the main objective(s) or question(s) the review addresses.	Yes
**METHODS**			
Eligibility criteria	3	Specify the inclusion and exclusion criteria for the review.	Yes
Information sources	4	Specify the information sources (e.g. databases, registers) used to identify studies and the date when each was last searched.	Yes
Risk of bias	5	Specify the methods used to assess risk of bias in the included studies.	Yes
Synthesis of results	6	Specify the methods used to present and synthesise results.	Yes
**RESULTS**			
Included studies	7	Give the total number of included studies and participants and summarise relevant characteristics of studies.	Yes
Synthesis of results	8	Present results for main outcomes, preferably indicating the number of included studies and participants for each. If meta-analysis was done, report the summary estimate and confidence/credible interval. If comparing groups, indicate the direction of the effect (i.e. which group is favoured).	Yes
**DISCUSSION**			
Limitations of evidence	9	Provide a brief summary of the limitations of the evidence included in the review (e.g. study risk of bias, inconsistency and imprecision).	Yes
Interpretation	10	Provide a general interpretation of the results and important implications.	Yes
**OTHER**			
Funding	11	Specify the primary source of funding for the review.	Yes
Registration	12	Provide the register name and registration number.	No

From: Page MJ, McKenzie JE, Bossuyt PM, Boutron I, Hoffmann TC, Mulrow CD, et al. The PRISMA 2020 statement: an updated guideline for reporting systematic reviews. BMJ 2021;372:n71. doi: 10.1136/bmj.n71. This work is licensed under CC BY 4.0. To view a copy of this license, visit https://creativecommons.org/licenses/by/4.0/
