# Supplementary material for: Functional regimes define soil microbiome response to environmental change
Source: Nature. 2025 Jul 16;644(8078):1028–38. doi: 10.1038/s41586-025-09264-9 (PMC12390847; doi:10.1038/s41586-025-09264-9)
Supplement: Supplementary file 6 — PRISMA report for meta-analysis of soil denitrification literature [file 41586_2025_9264_MOESM6_ESM.pdf]

Meta-analysis of electron acceptor dynamics during denitrification in soil microcosms

Abstract

Background: Denitrification by bacteria in soils is critical for nitrogen cycling and greenhouse gas emissions. However, variations in the soil types and experimental conditions across studies have led to differing conclusions about how microbial denitrification is influenced by environmental conditions. pH and carbon availability are the chief environmental correlates with denitrification rates.

Objectives: The objective is to test the hypothesis that functional regimes described by differing rates and dynamics of nitrate utilization across pH conditions are evident in other studies using soil microcosms. Functional regimes are characterized by the values of two parameters for a model of community metabolism: indigenous biomass activity and available limiting nutrients.

Methods: Due to the breadth of denitrification literature, and the fact that including a study in our metanalysis required dynamic measurements of either abundances or metabolites, we were unable to use general search terms to define a reasonably sized pool of potential studies. Instead, we performed a search based on forward/backward citation searches from an inclusive library of the key papers in the field. Inclusion criteria required presence of quantitative measurements of denitrification rates or metabolite dynamics, soil pH, and metadata (C/N ratio, soil particle composition). The risk of bias was assessed via how well soils spanned the soil texture (particle composition) classification triangle (**Fig. 6A**).

Results: Our meta-analysis confirmed that distinct functional regimes (Regime II and III) exist in diverse soils. Across soils with different native pH levels, the transition from Regime II to Regime III during basic perturbations was observed (**Fig. 6C**) In addition, Denitrification enzyme activity declined under strong pH perturbations across multiple independent datasets, supporting the pattern of reduced biomass activity in Regimes I and III (**Fig. 6F**). Nitrogen utilization dynamics revealed both the linearity near native pH and increase in reduction rates upon carbon amendment, mirroring the behavior predicted in Regime II (**Fig. 6G**).

Discussion: Our re-analysis of historical datasets revealed consistent functional regimes despite differences in soil type and experimental design, supporting the generality of our framework. The separation of early and late denitrification rates allowed us to infer the two parameters of our model. These findings demonstrate that functional regimes during pH perturbation are robust and observable in legacy datasets.

Funding: This work was supported by the National Science Foundation Division of Emerging Frontiers EF 2025293 (S.K.) and EF 2025521 (M.M.) and by National Science Foundation PHY 2310746 (M.T.). S.K. acknowledges the National Institute of General Medical Sciences R01GM151538, and support from the National Science Foundation through the Center for Living Systems (grant no. 2317138). S.K. and M.T. acknowledge CAREER awards from the

National Science Foundation (BIO/MCB 2340416 and PHY-2340791). S.K. and M.M. acknowledge financial support from the National Institute for Mathematics and Theory in Biology (Simons Foundation award MP-TMPS-00005320 and National Science Foundation award DMS-2235451). MM was supported by The National Science Foundation-Simons Center for Quantitative Biology at Northwestern University and the Simons Foundation grant 597491. MM is a Simons Investigator. This project has been made possible in part by grant number DAF2023-329587 from the Chan Zuckerberg Initiative DAF, an advised fund of the Silicon Valley Community Foundation. Any opinions, findings, conclusions, or recommendations expressed in this material are those of the authors and do not necessarily reflect the views of the National Science Foundation.

Introduction

Rationale: While many studies have examined denitrification in soils, differences in soil types and experimental designs have led to varying interpretations of how microbial communities utilize nitrate in response to changing environmental conditions like pH and nutrient availability. Revisiting these studies through a common mechanistic lens may reveal consistent patterns that were not previously apparent.

Objectives: This meta-analysis aims to assess whether the functional regimes identified in our framework are also present in historical denitrification studies. Rather than exhaustively surveying the literature, we focus on re-analyzing representative time-series datasets to validate the robustness and generality of our proposed mechanistic regimes.

Methods

Eligibility criteria: We included studies that reported quantitative measurements of denitrification rates or nitrogen compound dynamics over time, along with metadata on pH, carbon content/amendment, and soil properties. We focused on studies with time-resolved data sufficient to evaluate functional regimes.

Information sources: We manually searched peer-reviewed articles using Google scholar and Web of Science.

Search strategy: No formal database filters or automation tools were used. Because the inclusion criteria were so stringent, we could not survey all denitrification-related literature. Instead we took a forward/backward citation search approach. We identified several key papers in the field and began searching every paper that was cited, or cited that paper. We seeded this search with three key papers. For any papers cited or that cited one of these papers, we examined the abstract and figures. Any papers with usable data then seeded the next round of search via citations and cited articles. This yielded approximately 15 papers that were considered in detail, 9 of which we were able to quantitatively analyze the available data. Approximately 200 papers were considered during this search.

Selection process: Studies were selected based on whether plots showed measurable denitrification dynamics (e.g., nitrate, nitrite, N_2O, N_2) with corresponding metadata. Authors

(S.K., K.K.L., S.L.) screened and selected the studies, focusing on diversity in soil type, geographic location, and experimental design.

Data collection process: Data were extracted manually from published figures via ImageJ. One author (S.L.) handled the digitization and metadata annotation for all studies. No contact with study authors was made for raw data retrieval.

Data items:
10a. Primary outcomes were denitrification enzyme activity (DEA), denitrification potential (DP), and time-resolved concentrations of nitrogen compounds, either nitrate or nitrous oxide. We used early- and late-stage rates for inferring microbial activity and nutrient limitation via our model.

10b. Additional variables included soil texture, native pH, treatment pH, carbon amendments, and C/N ratio. When metadata were missing, we inferred approximate values based on descriptions in the methods sections or accompanying tables. For example, if clay:silt:sand ratios were not published, but a soil type name was published, we took median values for the clay:silt:sand ratios for that soil type.

Study risk of bias assessment: We minimized the risk of missing potential studies via the repeated seeding of our branching search process. We assessed the risk that our scientific findings were biased by the representativeness of the soil types (triangular soil texture classification in **Fig. 6A**).

Effect measures:
We did not compute traditional effect sizes. Instead, we used slopes of early and late denitrification phases to infer microbial parameters (e.g., $\tilde{x}(0)$, $\gamma\tilde{C}(0)$) and tracked directional changes across pH and carbon perturbation gradients. There was no statistical test performed.

Synthesis methods:
13a. We selected studies that had sufficient temporal resolution and metadata to allow inference of functional parameters and mapping to our regime framework.
13b. When necessary, we converted visual data into numerical form by digitizing plots. Early linear fits were used for $\tilde{x}(0)$; the maximum slope of non-linear curves was used for $\gamma\tilde{C}(0)$.
13c. Results were displayed using scatter plots (e.g., **Fig. 6C, F, G**), and additional metadata were compiled in **Table S2**.
13d. No formal statistical meta-analysis was conducted. We used qualitative synthesis of rate comparisons across regimes to assess consistency with our theoretical predictions. As claimed in the manuscript, the conservation of regimes is a qualitative result.
13e. Soil type, native pH, and treatment conditions were used to explain heterogeneity in observed patterns.
13f. Sensitivity was checked by comparing patterns across subsets of studies, and no qualitative differences were observed.

Reporting bias assessment: We acknowledge a likely reporting bias toward studies that measured denitrification dynamics or had sufficient perturbation conditions.

Certainty assessment: Certainty in our findings was evaluated based on the consistency of observed patterns across independent studies and parameter inference across various soil types. Qualitative agreement across studies increases confidence in the generality of the regimes.

Results

Study Selection

16a. Our meta-analysis identified 9 representative denitrification studies spanning 70 years (1956-2024) that met our inclusion criteria. These studies provided 19 soil samples with time-series measurements of denitrification dynamics under pH-perturbed or carbon-amended conditions.

16b. Several studies that appeared relevant were excluded because they lacked either (1) quantitative time-series measurements of denitrification-related metabolites, (2) sufficient metadata about soil properties (pH, C/N ratio, particle composition), or (3) clear experimental conditions for pH or carbon perturbations. These exclusions ensured only studies with interpretable dynamics and complete metadata were analyzed.

Study Characteristics:

The included studies represented 19 diverse soils:

- Anderson et al. (2018): 1 New Zealand soil (agriculture)
- Šimek et al. (2002): 5 Czech soils (arable land, grassland)
- Parkin et al. (1985): 2 Michigan soils (agriculture)
- Nömmik (1956): 2 Norwegian soils (unknown land use)
- Bremner and Shaw (1958a): 2 UK soils (arable)
- Bremner and Shaw (1958b): 2 UK soils (pasture)
- Šimek & Hopkins (1999): 1 Czech soil (arable)
- Khalifa & Folz (2024): 4 Oklahoma soils (grassland)

These covered silty clay loams, sandy loams, and clay soils across Europe and North America (**Fig. 6A**, **Table S2**).

Risk of Bias: The risk of bias was assessed based on soil representativeness (covering the soil texture classification triangle) and completeness of metadata (pH, C/N, particle composition). All included studies may contain bias. Studies occasionally missed C/N data, land usage or approximate geolocations.

Results of Individual Studies:

- Anderson et al. (2018): Confirmed enrichment of Bacillota in basic perturbations (**Fig. 6E**)
- Šimek et al. (2002): DEA/DP measurements evidencing Regime I, II, and III (**Fig. 6C**)
- Parkin et al. (1985): Indigenous biomass activity decreases in extreme perturbations both in acid/neutral soil (**Fig. 6F**)
- Nömmik (1956): Linear dynamics found in Regime II (**Fig. 6G** right)
- Bremner and Shaw (1958): Linear dynamics found in Regime II and transition to Regime III due to carbon amendments (**Fig. 6G** left)

- Šimek & Hopkins (1999): Indigenous biomass activity decreases in extreme perturbations both in acid/neutral soil (**Fig. 6F**)
- Khalifa & Folz (2024): Indigenous biomass activity decreases in extreme perturbations both in acid/neutral soil (**Fig. 6F**)

Synthesis Results:
20a. The nine studies consistently showed (1) Regime II (linear dynamics near native pH) and (2) Regime III during basic pH perturbations. Fewer studies showed Regime I due to a lack of data without antibiotics and acidic pH perturbations.
20b. We did not statistically synthesize the denitrification data from different soils. Instead, we show for individual soils the directional changes across pH and carbon perturbation gradients (**Fig. 6**).
20cd. Soil type and different experimental conditions may have influenced the denitrification dynamics. However, the qualitative behavior of distinct soils reflected functional regimes.

Reporting Biases: Potential biases could be the overrepresentation of European soils and the exclusion of studies without time-series data and limited tropical/arid soil coverage.

Certainty of Evidence: High certainty exists for functional Regime II and Regime III and carbon limitation in Regime II. Limited evidence in these studies is available for Regime I since only one study showed this regime. Limitations include methodological heterogeneity and small sample size (soil n=19).

Discussion

23a. The consistent observation of functional regimes across diverse soils and experimental conditions suggests these regimes are not only confined to Cook Agronomy Farm soils but are generalizable to other soils.
23b. While consistent within the analyzed dataset, the evidence in literature remains limited by the handful number of dynamic measurements available, heterogeneous methodologies across studies, and underrepresentation of certain soil types (e.g., tropical, arid).
23c. Our review process prioritized studies with time-series data, potentially excluding relevant static measurements, and relied on digitized plots where raw data were unavailable.
23d. These findings highlight the importance of making dynamic measurements in future denitrification studies.

Other information

Registration and Protocol: This meta-analysis was not formally registered other than being part of the manuscript, and no protocol was prepared prior to analysis.

Support: Funding was provided by NSF, NIH, Simons Foundation, and CZI grants; all funders had no role in study design or interpretation.

Competing Interests: The authors declare no financial conflicts.

Availability of Materials: Metadata table (**Table S2**) is provided in supplementary files. The data and analysis code are deposited at the [Open Science Framework](https://osf.io/ctf8k/) (https://osf.io/ctf8k/).

Section	Item #	Checklist item	Location where item is reported
**TITLE**			
Title	1	Identify the report as a systematic review.	Page 1
**ABSTRACT**			
Abstract	2	See the PRISMA 2020 for Abstracts checklist.	Page 1
**INTRODUCTION**			
Rationale	3	Describe the rationale for the review in the context of existing knowledge.	Page 2
Objectives	4	Provide an explicit statement of the objective(s) or question(s) the review addresses.	Page 2
**METHODS**			
Eligibility criteria	5	Specify the inclusion and exclusion criteria for the review and how studies were grouped for the syntheses.	Page 2
Information sources	6	Specify all databases, registers, websites, organisations, reference lists and other sources searched or consulted to identify studies. Specify the date when each source was last searched or consulted.	Page 2
Search strategy	7	Present the full search strategies for all databases, registers and websites, including any filters and limits used.	Page 2
Selection process	8	Specify the methods used to decide whether a study met the inclusion criteria of the review, including how many reviewers screened each record and each report retrieved, whether they worked independently, and if applicable, details of automation tools used in the process.	Page 2
Data collection process	9	Specify the methods used to collect data from reports, including how many reviewers collected data from each report, whether they worked independently, any processes for obtaining or confirming data from study investigators, and if applicable, details of automation tools used in the process.	Page 3
Data items	10a	List and define all outcomes for which data were sought. Specify whether all results that were compatible with each outcome domain in each study were sought (e.g. for all measures, time points, analyses), and if not, the methods used to decide which results to collect.	Page 3
	10b	List and define all other variables for which data were sought (e.g. participant and intervention characteristics, funding sources). Describe any assumptions made about any missing or unclear information.	Page 3
Study risk of bias assessment	11	Specify the methods used to assess risk of bias in the included studies, including details of the tool(s) used, how many reviewers assessed each study and whether they worked independently, and if applicable, details of automation tools used in the process.	Page 3
Effect measures	12	Specify for each outcome the effect measure(s) (e.g. risk ratio, mean difference) used in the synthesis or presentation of results.	Page 3
Synthesis methods	13a	Describe the processes used to decide which studies were eligible for each synthesis (e.g. tabulating the study intervention characteristics and comparing against the planned groups for each synthesis (item #5)).	Page 3
	13b	Describe any methods required to prepare the data for presentation or synthesis, such as handling of missing summary statistics, or data conversions.	Page 3
	13c	Describe any methods used to tabulate or visually display results of individual studies and syntheses.	Page 3
	13d	Describe any methods used to synthesize results and provide a rationale for the choice(s). If meta-analysis was performed, describe the model(s), method(s) to identify the presence and extent of statistical heterogeneity, and software package(s) used.	Page 3
	13e	Describe any methods used to explore possible causes of heterogeneity among study results (e.g. subgroup analysis, meta-regression).	Page 3
	13f	Describe any sensitivity analyses conducted to assess robustness of the	Page 3

Section	Item #	Checklist item	Location where item is reported
		synthesized results.	
Reporting bias assessment	14	Describe any methods used to assess risk of bias due to missing results in a synthesis (arising from reporting biases).	Page 3
Certainty assessment	15	Describe any methods used to assess certainty (or confidence) in the body of evidence for an outcome.	Page 4
**RESULTS**			
Study selection	16a	Describe the results of the search and selection process, from the number of records identified in the search to the number of studies included in the review, ideally using a flow diagram.	Page 4
	16b	Cite studies that might appear to meet the inclusion criteria, but which were excluded, and explain why they were excluded.	Page 4
Study characteristics	17	Cite each included study and present its characteristics.	Page 4
Risk of bias in studies	18	Present assessments of risk of bias for each included study.	Page 4
Results of individual studies	19	For all outcomes, present, for each study: (a) summary statistics for each group (where appropriate) and (b) an effect estimate and its precision (e.g. confidence/credible interval), ideally using structured tables or plots.	Page 4
Results of syntheses	20a	For each synthesis, briefly summarise the characteristics and risk of bias among contributing studies.	Page 5
	20b	Present results of all statistical syntheses conducted. If meta-analysis was done, present for each the summary estimate and its precision (e.g. confidence/credible interval) and measures of statistical heterogeneity. If comparing groups, describe the direction of the effect.	Page 5
	20c	Present results of all investigations of possible causes of heterogeneity among study results.	Page 5
	20d	Present results of all sensitivity analyses conducted to assess the robustness of the synthesized results.	Page 5
Reporting biases	21	Present assessments of risk of bias due to missing results (arising from reporting biases) for each synthesis assessed.	Page 5
Certainty of evidence	22	Present assessments of certainty (or confidence) in the body of evidence for each outcome assessed.	Page 5
**DISCUSSION**			
Discussion	23a	Provide a general interpretation of the results in the context of other evidence.	Page 5
	23b	Discuss any limitations of the evidence included in the review.	Page 5
	23c	Discuss any limitations of the review processes used.	Page 5
	23d	Discuss implications of the results for practice, policy, and future research.	Page 5
**OTHER INFORMATION**			
Registration and protocol	24a	Provide registration information for the review, including register name and registration number, or state that the review was not registered.	Page 5
	24b	Indicate where the review protocol can be accessed, or state that a protocol was not prepared.	Page 5
	24c	Describe and explain any amendments to information provided at registration or in the protocol.	Page 5
Support	25	Describe sources of financial or non-financial support for the review, and the role of the funders or sponsors in the review.	Page 5
Competing interests	26	Declare any competing interests of review authors.	Page 5
Availability of data, code and other materials	27	Report which of the following are publicly available and where they can be found: template data collection forms; data extracted from included studies; data used for all analyses; analytic code; any other materials used in the	Page 5

Section	Item #	Checklist item	Location where item is reported
		review.	

Abstract checklist

Section and Topic	Item #	Checklist item	Reported (Yes/No)
**TITLE**			
Title	1	Identify the report as a systematic review.	Yes
**BACKGROUND**			
Objectives	2	Provide an explicit statement of the main objective(s) or question(s) the review addresses.	Yes
**METHODS**			
Eligibility criteria	3	Specify the inclusion and exclusion criteria for the review.	Yes
Information sources	4	Specify the information sources (e.g. databases, registers) used to identify studies and the date when each was last searched.	Yes
Risk of bias	5	Specify the methods used to assess risk of bias in the included studies.	Yes
Synthesis of results	6	Specify the methods used to present and synthesise results.	Yes
**RESULTS**			
Included studies	7	Give the total number of included studies and participants and summarise relevant characteristics of studies.	Yes
Synthesis of results	8	Present results for main outcomes, preferably indicating the number of included studies and participants for each. If meta-analysis was done, report the summary estimate and confidence/credible interval. If comparing groups, indicate the direction of the effect (i.e. which group is favoured).	Yes
**DISCUSSION**			
Limitations of evidence	9	Provide a brief summary of the limitations of the evidence included in the review (e.g. study risk of bias, inconsistency and imprecision).	Yes
Interpretation	10	Provide a general interpretation of the results and important implications.	Yes
**OTHER**			
Funding	11	Specify the primary source of funding for the review.	Yes
Registration	12	Provide the register name and registration number.	No

From: Page MJ, McKenzie JE, Bossuyt PM, Boutron I, Hoffmann TC, Mulrow CD, et al. The PRISMA 2020 statement: an updated guideline for reporting systematic reviews. BMJ 2021;372:n71. doi: 10.1136/bmj.n71. This work is licensed under CC BY 4.0. To view a copy of this license, visit https://creativecommons.org/licenses/by/4.0/
